# A Comprehensive Genomic Analysis Reveals the Genetic Landscape of Mitochondrial Respiratory Chain Complex Deficiencies

**DOI:** 10.1371/journal.pgen.1005679

**Published:** 2016-01-07

**Authors:** Masakazu Kohda, Yoshimi Tokuzawa, Yoshihito Kishita, Hiromi Nyuzuki, Yohsuke Moriyama, Yosuke Mizuno, Tomoko Hirata, Yukiko Yatsuka, Yzumi Yamashita-Sugahara, Yutaka Nakachi, Hidemasa Kato, Akihiko Okuda, Shunsuke Tamaru, Nurun Nahar Borna, Kengo Banshoya, Toshiro Aigaki, Yukiko Sato-Miyata, Kohei Ohnuma, Tsutomu Suzuki, Asuteka Nagao, Hazuki Maehata, Fumihiko Matsuda, Koichiro Higasa, Masao Nagasaki, Jun Yasuda, Masayuki Yamamoto, Takuya Fushimi, Masaru Shimura, Keiko Kaiho-Ichimoto, Hiroko Harashima, Taro Yamazaki, Masato Mori, Kei Murayama, Akira Ohtake, Yasushi Okazaki

**Affiliations:** 1 Division of Translational Research, Research Center for Genomic Medicine, Saitama Medical University, Hidaka-shi, Saitama, Japan; 2 Division of Functional Genomics & Systems Medicine, Research Center for Genomic Medicine, Saitama Medical University, Hidaka-shi, Saitama, Japan; 3 Division of Developmental Biology, Research Center for Genomic Medicine, Saitama Medical University, Hidaka-shi, Saitama, Japan; 4 Chemicals Assessment and Research Center, Chemicals Evaluation and Research Institute, Japan (CERI), Sugito-machi, Kitakatsushika-gun, Saitama, Japan; 5 Department of Biological Sciences, Tokyo Metropolitan University, Hachioji, Tokyo, Japan; 6 Department of Chemistry and Biotechnology, Graduate School of Engineering, University of Tokyo, Bunkyo-ku, Tokyo, Japan; 7 Center for Genomic Medicine, Kyoto University Graduate School of Medicine, Sakyo-ku, Kyoto, Japan; 8 Department of Integrative Genomics, Tohoku Medical Megabank Organization, Tohoku University, Aoba-ku, Sendai, Miyagi, Japan; 9 Graduate School of Medicine, Tohoku University, Aoba-ku, Sendai, Miyagi, Japan; 10 Graduate School of Information Sciences, Tohoku University, Sendai, Miyagi, Japan; 11 Department of Metabolism, Chiba Children's Hospital, Midori, Chiba, Japan; 12 Department of Pediatrics, Saitama Medical University, Moroyama-machi, Iruma-gun, Saitama, Japan; 13 Department of Pediatrics, Matsudo City Hospital, Matsudo-shi, Chiba, Japan; Stanford University School of Medicine, UNITED STATES

## Abstract

Mitochondrial disorders have the highest incidence among congenital metabolic disorders characterized by biochemical respiratory chain complex deficiencies. It occurs at a rate of 1 in 5,000 births, and has phenotypic and genetic heterogeneity. Mutations in about 1,500 nuclear encoded mitochondrial proteins may cause mitochondrial dysfunction of energy production and mitochondrial disorders. More than 250 genes that cause mitochondrial disorders have been reported to date. However exact genetic diagnosis for patients still remained largely unknown. To reveal this heterogeneity, we performed comprehensive genomic analyses for 142 patients with childhood-onset mitochondrial respiratory chain complex deficiencies. The approach includes whole mtDNA and exome analyses using high-throughput sequencing, and chromosomal aberration analyses using high-density oligonucleotide arrays. We identified 37 novel mutations in known mitochondrial disease genes and 3 mitochondria-related genes (*MRPS23*, *QRSL1*, and *PNPLA4*) as novel causative genes. We also identified 2 genes known to cause monogenic diseases (*MECP2* and *TNNI3*) and 3 chromosomal aberrations (6q24.3-q25.1, 17p12, and 22q11.21) as causes in this cohort. Our approaches enhance the ability to identify pathogenic gene mutations in patients with biochemically defined mitochondrial respiratory chain complex deficiencies in clinical settings. They also underscore clinical and genetic heterogeneity and will improve patient care of this complex disorder.

## Author Summary

Mitochondria play a crucial role in ATP biosynthesis and comprise proteins encoded in both the nuclear and mitochondrial genomes. Although more than 250 mitochondrial disease-causing genes have been reported, the exact genetic causes in patients remain largely unknown. Here, we aimed to provide further insights into the pathogenic mechanisms of mitochondrial disorders. We investigated the genes encoded in the nuclear and mitochondrial genomes using comprehensive genomic analysis in 142 patients with mitochondrial respiratory chain complex deficiencies. We identified 3 novel disease-causing mitochondria-related genes (*MRPS23*, *QRSL1*, and *PNPLA4*) as well as other disease-causing genes and novel pathogenic mutations in known mitochondrial disease-causing genes. All pathogenic mutations in this study are validated by genetic and/or functional evidence. Our findings, including the achievement of firm genetic diagnoses for 49 of 142 patients (34.5%), were higher than the general diagnosis rate of approximately 25% and demonstrated the value of comprehensive genomic analysis. Accordingly, we have shed light on the genetic heterogeneity underlying mitochondrial disorders.

## Introduction

Human oxidative phosphorylation (OXPHOS) disease has the highest incidence among congenital metabolic disorders characterized by a biochemical respiratory chain complex deficiencies and is thought to occur at a rate of 1 in 5,000 births[1]. No more than 15–30% of pediatric diseases diagnosed as mitochondrial disorders show mitochondrial DNA (mtDNA) abnormalities[2,3]; the remaining cases occur because of defects in genes encoded in the nucleus. A certain amount of nuclear-encoded gene products are present in the mitochondria, and roughly 1,500 are thought to play important roles in mitochondrial function[4,5].

It is particularly difficult to diagnose patients with OXPHOS disease at the molecular level because of the massive numbers of potentially involved nuclear genes and genes not yet linked to human disease. Therefore, identification of the causative genes and an understanding of the pathogenic mechanisms of OXPHOS disease remain unsolved challenges.

Recent studies[6,7] have shown that heterogeneous genetic backgrounds as well as genes previously not linked to mitochondrial functions or localization are associated with this disease. However, because of phenotypic and locus heterogeneity, only a fraction of patients has been identified to date. Limitations in target resequencing have motivated us to apply a comprehensive genomic analysis for more accurate molecular diagnosis and for the identification of novel causative genes.

Here, we aimed to determine whether a comprehensive genomic analysis approach could be used to reveal the broad spectrum of genetic background of the disease[8]. One hundred and forty-two unrelated individuals with displayed childhood-onset mitochondrial respiratory chain complex deficiencies were selected. We applied long-range polymerase chain reaction (PCR)-based whole mtDNA sequencing, whole exome sequencing (WES), and high-density oligonucleotide arrays to identify single-nucleotide variants (SNVs), small insertions or deletions (indels), and chromosomal aberrations for comprehensive genomic analyses.

## Results

### Comprehensive genomic analysis in 142 patients

**Fig 1:**
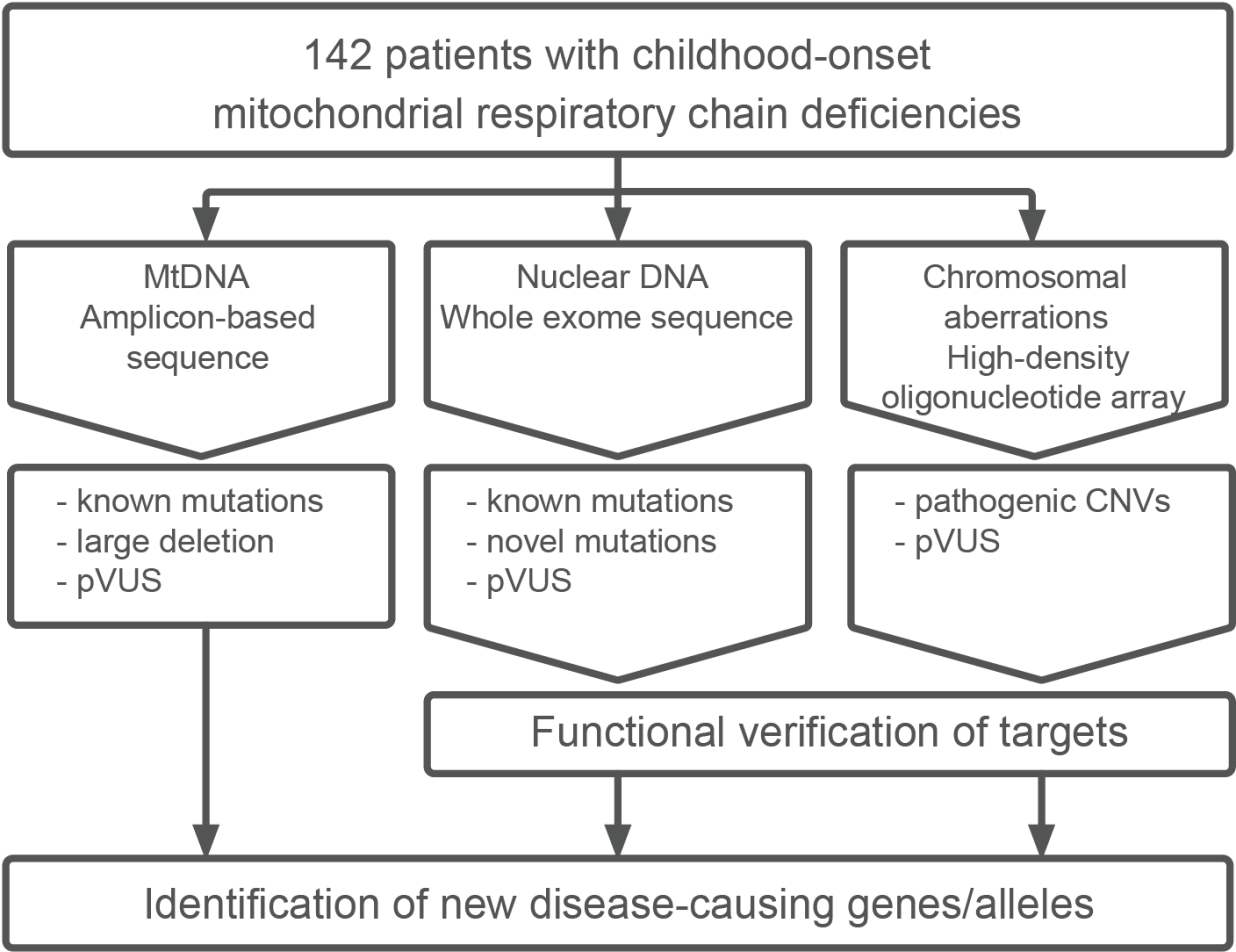
Schematic of comprehensive genomic analysis of 142 patients. All 142 patients were subjected to mtDNA amplicon-based sequencing, WES, and high-density oligonucleotide array analysis in parallel. Variants were filtered on the basis of their rarity in public databases and population-matched datasets. For each analysis, candidate variants were prioritized on the basis of the type of variant. Candidate variants were validated by Sanger sequencing and tested for segregation within the family if DNA was available. mtDNA, mitochondrial DNA; pVUS, prioritized variant of unknown significance; CNV, copy number variation

In this study, 142 patients with childhood-onset mitochondrial respiratory chain complex deficiencies were enrolled and subjected to comprehensive genomic analyses (detailed clinical characteristics are described in S1 Table). A schematic workflow of these analyses is shown in Fig 1. Comprehensive genomic analyses included three approaches: (i) amplicon-based whole mtDNA sequencing for pathogenic mutations and large duplications/deletions, (ii) WES for pathogenic mutations in nuclear DNA, and (iii) high-density oligonucleotide arrays for chromosomal aberrations. The prioritized variants derived from each approach are described below.

### Prioritized variants in 142 patients

After comprehensive genomic analysis shown in Fig 1, rare variants were filtered out and prioritized on the basis of the strategy described below. For mtDNA variants, we targeted variants confirmed and reported in MITOMAP[9]. Exome sequencing covered 89% (ranged: from 70%–to 98%) of the targeted bases, with more than 20-fold coverage. Detailed sequence statistics is shown in S2 Table.

The precise strategy for WES variant prioritization is shown in S2 Fig. We evaluated our prioritization pipeline to validate whether it could feasibly enrich known OXPHOS disease-causing genes or mitochondria-related genes (S3 Fig). Known OXPHOS disease-causing genes were clearly enriched in disease cases, whereas no prioritized genes were detected in healthy controls. Compared with healthy controls, mitochondria-related genes also exhibited a 1.64-fold enrichment. No enrichment was observed in randomly selected genes. These results suggest that mitochondria-related gene enrichment is caused by unidentified causative genes.

To analyze chromosomal aberrations, we focused on rather large (>100 Kb) deletions or duplications. For prioritizing candidate aberrations, we filtered out deleted or duplicated regions found in the 524 in-house controls and manually curated the pathogenicity of the aberrations by referring to the OMIM, DGV, and DECIPHER databases.

**Fig 2:**
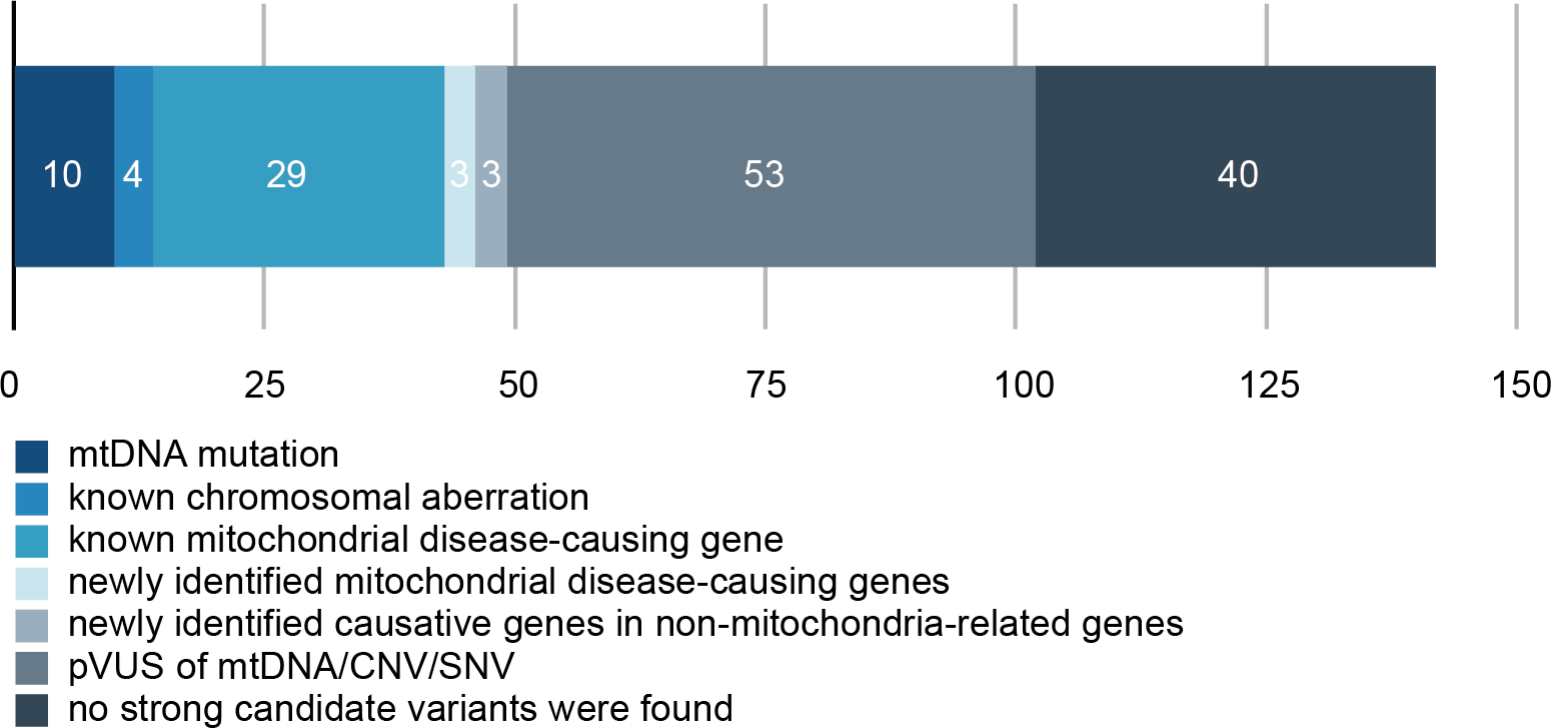
Breakdown of 142 patients categorized by the type of mutations/variants in comprehensive genomic analysis. Numerals in each colored box indicate the number of patients. Patients harboring multiple variants were assigned to each box on the basis of the highest priority variant in all cases.

A breakdown of the 142 patients according to prioritized variants is shown in Fig 2. Of the 142 patients with mitochondrial respiratory chain complex deficiencies, 102 (71.8%) harbored at least 1 prioritized mtDNA mutation, nuclear gene mutation, or chromosomal abnormality. Ten (7.0%) patients harbored mtDNA mutations, including one large deletion (S4 Fig). In 29 patients (20.4%), firm molecular diagnoses were made in 20 genes previously linked to mitochondrial disorders. We newly confirmed 3 mitochondria-related genes (*MRPS23*, *QRSL1*, and *PNPLA4*) as causative genes of mitochondrial respiratory chain complex deficiencies. Three patients (2.1%) harbored mutations in genes known to cause monogenic diseases (*MECP2* and *TNNI3*). Intriguingly, 4 patients (2.8%) had pathogenic chromosomal deletions previously linked to other disorders (6q24.3-q25.1, 22q11.21, and 17p12) but not linked to mitochondrial respiratory chain complex deficiencies.

In 53 (37.3%) patients, we identified and designated candidate genes or loci as prioritized variants of unknown significance (pVUS) because these variants have possibilities to be pathogenic but have insufficient evidence to support a disease linage. The current lack of functional validation for linking these genes with mitochondrial disorders led us to classify these variants as inconclusive with respect to pathogenicity (S3, S4 and S5 Tables). The remaining 40 (28.2%) patients lacked prioritized nuclear variants, mtDNA variants, and chromosomal abnormalities.

### New genetic diagnoses for cases with previously established nuclear disease genes

Twenty-two genes were prioritized in 31 patients (Table 1). Of these, 29 patients harbored 41 disease-causing mutations in 20 genes known to cause OXPHOS disease: *ACAD9*, *BOLA3*, *COQ4*, *COX10*, *EARS2*, *ECHS1*, *GFM1*, *GTPBP3*, *KARS*, *MPV17*, *NDUFA10*, *NDUFAF6*, *NDUFB11*, *NDUFS4*, *RARS2*, *RRM2B*, *SCO2*, *SUCLA2*, *TAZ*, and *TUFM*. All such mutations were confirmed through Sanger sequencing and haplotype phasing. In particular, 8 patients had homozygous mutations, 19 had compound heterozygous mutations, and 2 had hemizygous mutations. Of the 41 mutations, 37 were novel and 4 were reported as pathogenic in the Human Gene Mutation Database[10] (HGMD, professional version 2013.10).

**Table 1:** New genetic diagnoses for cases with previously established nuclear disease genes.

ID	Clinical diagnosis	Complex type	Genetic diagnosis	Gene	Variations	Supporting evidence
Pt090	NLIMD	CI	Firm	*ACAD9* (NM_014049)	c.1150G>A:p.V384M, c.1817T>A:p.L606H	Segregation
Pt025	NLIMD	CI	Firm	*ACAD9* (NM_014049)	c.811T>G:p.C271G, c.1766-2A>G	Rescue
Pt045	LIMD	CC	Firm	*BOLA3* (NM_212552)	c.225_229del:p.K75fs, c.287A>G:p.H96R	Shared rare variant in four patients, rescue
Pt268	LIMD	CC	Firm	*BOLA3* (NM_212552)	c.287A>G:p.H96R, c.287A>G:p.H96R	Shared rare variant in four patients, rescue
Pt286	LD	CC	Firm	*BOLA3* (NM_212552)	c.287A>G:p.H96R, c.287A>G:p.H96R	Shared rare variant in four patients, rescue
Pt314	CM	CC	Firm	*BOLA3* (NM_212552)	c.287A>G:p.H96R, c.287A>G:p.H96R	Shared rare variant in four patients, rescue
Pt113	LIMD	CC	Firm	*COQ4* (NM_016035)	c.718C>T:p.R240C, c.421C>T:p.R141X	Rescue; patient is included in the study by Brea-Calvo et al[11]
Pt223	CM	CC	Firm	*COX10* (NM_001303)	c.862G>A:p.G288R, c.1259C>T:p.P420L	Rescue
Pt691	ND	CIV	Firm	*EARS2* (NM_001083614)	c.319C>T:p.R107C, c.1466G>A:p.R489Q	*De novo* (c.319C>T)
Pt346	LIMD	CI	Firm	*ECHS1* (NM_004092)	c.176A>G:p.N59S, c.476A>G:p.Q159R	Known; patient is included in the study by Haack et al[12]
Pt376	LD	CIV	Firm	*ECHS1* (NM_004092)	c.98T>C:p.F33S, c.176A>G:p.N59S	Known; patient is included in the study by Haack et al[12]
Pt112	HD	CC	Firm	*GFM1* (NM_024996)	c.170C>A:p.S57Y, c.748C>T:p.R250W	Known[13]
Pt751	LD	CC	Firm	*GTPBP3* (NM_032620)	c.8G>T:p.R3L, c.923-947del (p.E309fs)	Patient is included in the study by Kopajtich R. et al[14]
Pt459	MC	CC	Firm	*KARS* (NM_005548)	c.1343T>A:p.V448D, c.953T>C:p.I318T	Rescue
Pt339	HD (MTDPS)	CC	Firm	*MPV17* (NM_002437)	c.293C>T:p.P98L, c.376-1G>A	Known[15], mtDNA decreased to 20.5% in hepatic tissue
Pt057	NLIMD	CI	Firm	*NDUFA10* (NM_004544)	c.383_384insTAA:p.S128delinsIS, c.881T>C:p.L294P	Rescue
Pt512	LD	CI	Firm	*NDUFAF6* (NM_152416)	c.226T>C:p.S76P, c.805C>G:p.H269D	Independent rare variant in two patients (c.805 C >G), rescue
Pt598	LD	CI	Firm	*NDUFAF6* (NM_152416)	c.206A>T:p.D69V, c.371T>C:p.I124T	Independent rare variant in two patients (c.371 T >C)
Pt101	LD	CI	Firm	*NDUFAF6* (NM_152416)	c.371T>C:p.I124T, c.805C>G:p.H269D	Independent rare variants in two patients (c.371T>C, c.805C>G), segregation
Pt330	MC	CI	Firm	*NDUFAF6* (NM_152416)	c.820A>G:p.R274G, c.820A>G:p.R274G	Rescue
Pt067	LIMD	CI	Firm	*NDUFB11* (NM_001135998)	c.361G>A:p.E121K (hemizygous)	Rescue, *de novo*, functional assay
Pt711	LD	CI	Firm	*NDUFS4* (NM_002495)	c.340T>C:p.W114R, c.340T>C:p.W114R	LCSH
Pt222	NLIMD	CIV	Firm	*RARS2* (NM_020320)	c.1321C>T:p.L441F, c.1306G>T:p.D436Y	Segregation
Pt652	MC	CC	Firm	*RRM2B* (NM_015713)	c.97C>T:p.P33S, c.97C>T:p.P33S	Segregation
Pt628	LD	CC	Firm	*SCO2* (NM_001169109)	c.577G>A:p.G193S, c.773T>C:p.M258T	Known[16], segregation
Pt105	MC	CIV	Firm	*SUCLA2* (NM_003850)	c.1048G>A:p.G350S, c.1048G>A:p.G350S	Known[17], LCSH, no deletion detected by array
Pt634	NLIMD	CC	Firm	*TAZ* (NM_000116)	c.36_57del:p.12_19del (hemizygous)	NDP
Pt559	NLIMD	CIV	Firm	*TUFM* (NM_003321)	c.440T>A:p.L147H, c.440T>A:p.L147H	Segregation
Pt622	LIMD	CC	Firm	*TUFM* (NM_003321)	c.440T>A:p.L147H, c.162delC:p.Y54X	Rescue, segregation
Pt550	LIMD (MTDPS)	CC	pVUS	*LRPPRC* (NM_133259)	c.1253A>C:p.N418T, c.2741C>A:p.P914Q	N.A.
Pt001	LIMD	CC	pVUS	*PC* (NM_022172)	c.1822G>A:p.G608R, c.2120C>T:p.T707M	N.A.

All listed variants were confirmed by Sanger sequencing of gDNA or cDNA. GERP scores of all listed variants, except for *EARS2* (c.319C>T), were >2.5. CI, complex I deficiency; CIV, complex IV deficiency; CC, combined complex deficiencies; CM, cardiomyopathy; HD, hepatic disease; LD, Leigh's disease; LIMD, lethal infantile mitochondrial disorder; MC, mitochondrial cytopathy; ND, neurodegenerative disorder; NLIMD, non-lethal infantile mitochondrial disorder; *de novo*, confirmed *de novo* variant by trio-based sequencing; known, known disease variant; NDP, no detectable protein; LCSH, long contiguous stretches of homozygosity from high-density oligonucleotide array; segregation, variant segregates with disease in family; N.A., not available.

*BOLA3*, which plays an essential role in iron–sulfur cluster production, was mutated in 4 unrelated patients with severe lactic acidosis and combined respiratory chain complex deficiencies (MIM 614299). Three of these patients (Pt045, Pt268, and Pt314) exhibited multiple organ failure; Pt268 and Pt314 had hypertrophic cardiomyopathy, and Pt045 developed seizures. All 4 patients exhibited decreased complex II activity and harbored the c.287A>G (p.H96R) mutation. Pt314 and Pt286 patients showed clear long contiguous stretches of homozygosity (LCSH) (2.8 Mb, 3.2 Mb respectively) around this p.H96R mutation. Pt268 also showed a short contiguous stretch of homozygosity (0.3 Mb). This homozygous region encompassing *BOLA3* was shared between these unrelated individuals. Sanger sequencing identified the parents for these three patients as heterozygous carriers of this mutation. No p.H96R carriers were found in NHLBI GO Exome Sequencing Project (ESP6500), and 1 Japanese carrier in 1000 Genomes Project (1KG) was found. We screened for mutations that violated the Hardy–Weinberg principle and only identified the p.H96R mutation. These results suggest that p.H96R is common in the Japanese population and has originated from a single founder (S5 and S6 Figs).

*NDUFAF6*, which plays an important role in complex I assembly, was mutated in 4 unrelated patients: Pt101, Pt512, and Pt598 exhibited regression, whereas Pt330 exhibited muscle atrophy. All patients had complex I deficiency (MIM 256000). Pt101 shared 1 allele with Pt512 and another with Pt598. Pt330 harbored homozygous mutation c.820A>G (p.R274G) located in 1.3 Mb LCSH. Sanger sequencing identified the parents as heterozygous carriers of this mutation. Only 1 family was reported to harbor a mutation in this gene[18] (S7, S8 and S9 Figs).

*NDUFB11*, recently reported as causative gene for microphthalmia with linear skin defects syndrome (MIM 300952) and encoding a complex I component, was mutated in Pt067, a boy born to non-consanguineous parents under conditions of intrauterine growth restriction; this patient presented with heart failure, respiratory failure, complex I deficiency, and lethal infantile mitochondrial disorder (LIMD). He harbored a hemizygous *de novo* mutation, c.361G>A (p.E121K), and there was no NDUFB11 protein expression in his fibroblasts (S10 Fig). Because the p.E121 residue is highly conserved in this gene, we performed functional *in vivo* assays using a *dndufb11*-knockdown *Drosophila* model (S11 Fig); compared with controls, the mean lifespan was significantly reduced and the metabolic rate was lower in knockdown flies. Blue-native polyacrylamide gel electrophoresis (BN-PAGE) analysis showed a loss of complex I assembly, and lactate and pyruvate levels were increased in the knockdown flies. The *in vivo dndufb11*-knockdown *Drosophila* experiment further supported this conclusion. While preparing this manuscript, two girls harboring mutations in *NDUFB11* with microphthalmia with linear skin defects were reported by van Rahden et al[19]. Our patient was a male and died 55 h after birth. He presented with redundant skin but had no linear skin defects.

Pt459, a boy with lactic acidosis, developmental delays, hypertrophic cardiomyopathy, seizure, and combined complex deficiencies (I and IV), harbored the compound heterozygous mutations c.1343T>A (p.V448D) and c.953T>C (p.I318T) in *KARS*. KARS is a lysyl-transfer RNA synthetase that produces 2 proteins that localize to the cytosol and mitochondria. A cDNA complementation assay revealed that mitochondrial KARS successfully rescued the enzyme defect, but not cytosolic form (S12 Fig). Detailed information and evidential support for other known genes are described in S1 Text.

### Newly identified mutations in mitochondria-related genes

Five (*MRPS23*, *C1QBP1*, *ALAS2*, *SLC25A26*, *QRSL1*) genes were identified as novel candidate genes (Tables 2 and S3). These genes were previously reported links to mitochondrial function but not mitochondrial respiratory chain complex deficiencies. Of these, we obtained pathogenic support for mutations in *MRPS23* and *QRSL1*. In addition, candidate genes that have no evidence of functional involvement in current mitochondrial biology are good targets for underlying novel mitochondrial biological functions. In one such case, we identified *PNPLA4* as a novel causative gene for mitochondrial respiratory chain complex deficiencies and proved its mitochondrial localization for the direct evidence of mitochondrial functions. The supportive evidence included (i) the identification of independent mutations in candidate genes in unrelated individuals with exquisitely similar phenotypes, (ii) rescue of patients’ cellular phenotypes in a cDNA complementation assay, and (iii) identification of a *de novo* mutation in the candidate gene. Other pVUS for candidate genes are shown in S3 Table.

**Table 2:** New genetic diagnoses for cases with genes not linked to mitochondrial respiratory chain complex deficiencies.

ID	Clinical diagnosis	Complex type	Genetic diagnosis	Gene	Variations	Supporting evidence
**Mitochondria-related genes**						
Pt276	HD	CC	Firm	*MRPS23* (NM_016070)	c.119C>G:p.P40R, c.119C>G:p.P40R	Rescue, functional assay, LCSH
Pt250	LIMD	CC	Firm	*QRSL1* (NM_018292)	c.398G>T:p.G133V, c.398G>T:p.G133V	Independent mutations in two patients, segregation, functional assay
Pt712	MC	CIV	Firm	*PNPLA4* (NM_001142389)	c.559C>T:p.R187X (hemizygous)	Mitochondrial localization, rescue, NDP
**Known to cause monogenic disease genes**						
Pt053	MC	CC	Firm	*MECP2* (NM_004992)	c.806delG:p.G269fs (hemizygous)	*De novo*, known[20]
Pt369	ND	CIV	Firm	*MECP2* (NM_001110792)	c.17_18insG:p.A6fs (hemizygous)	*De novo*
Pt827	CM	CI	Firm	*TNNI3* (NM_000363)	c.575G>A:p.R192H (heterozygous)	*De novo*, known[21]

All listed variants were confirmed by Sanger sequencing of gDNA or cDNA. GERP scores of all listed variants were >2.5. CM, cardiomyopathy; EP, enteropathy; HD, hepatic disease; LD, Leigh's disease; LIMD, lethal infantile mitochondrial disorder; MC, mitochondrial cytopathy; ND, neurodegenerative disorder; CI, complex I deficiency; CIV, complex IV deficiency; CC, combined complex deficiencies; *de novo*, confirmed *de novo* mutation by trio-based sequencing; known, known disease variant; NDP, no detectable protein; LCSH, long contiguous stretches of homozygosity from high-density oligonucleotide array; segregation, variant segregates with disease in family; splice, splicing defect observed in subject fibroblast cDNA; N.A., not available.

**Fig 3:**
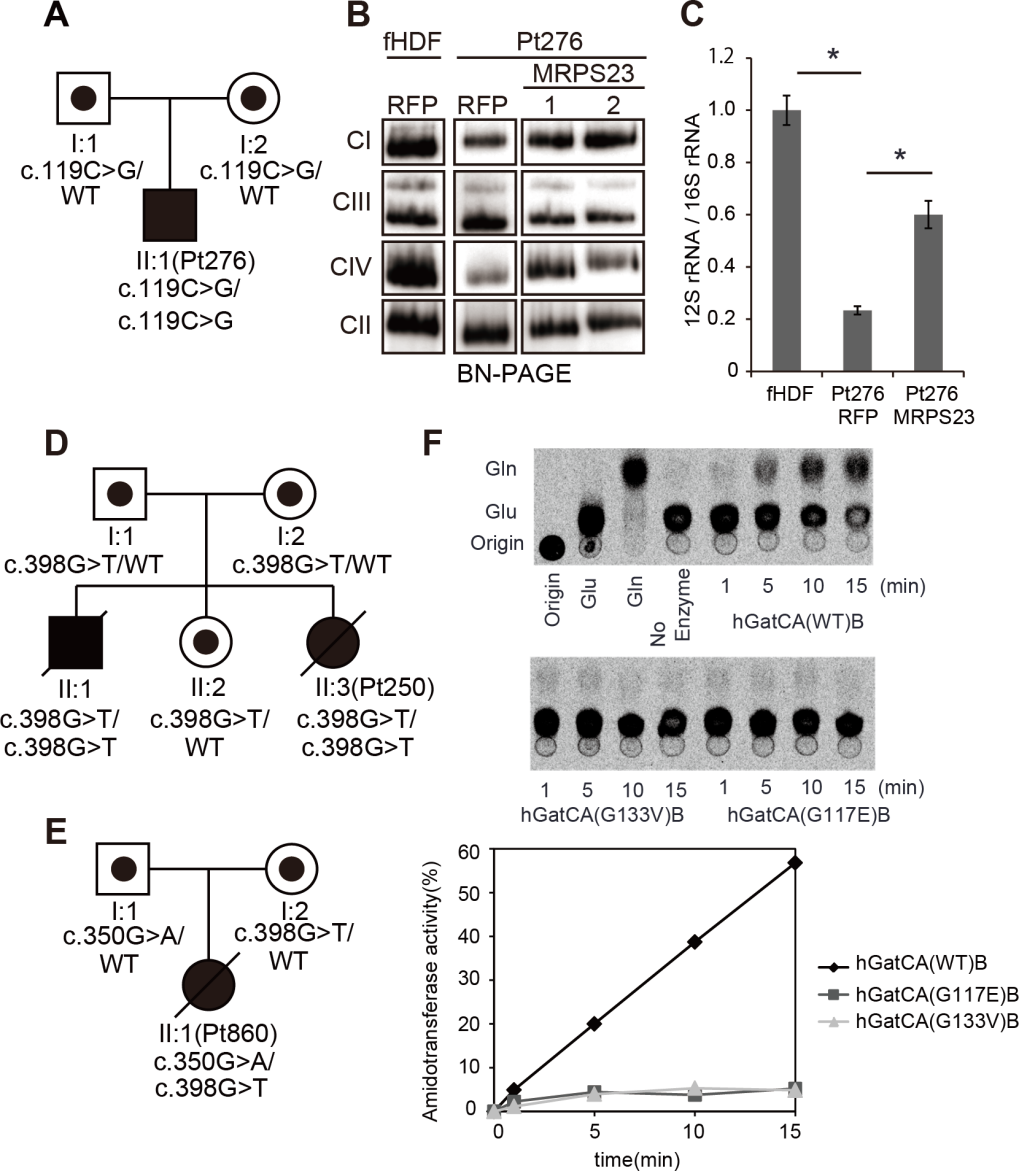
Newly identified causative genes via whole-exome sequencing analysis. (**A**) Family pedigrees of Pt276. (**B** and **C**) Complementation assay in Pt276 fibroblasts and normal control (fHDF). Mitochondrial fractions were isolated from fibroblasts with established stable expression of MRPS23 using a lentiviral expression system. Assembly levels and 12s rRNA stability were compared between control and *MRPS23* expressing fibroblasts by BN-PAGE /Western blotting (**B**) and qRT-PCR (**C**). fHDF: normal fetal human dermal fibroblast, RFP: *mito-TurboRFP-V5*, MRPS23: *MRPS23-V5*. Significance was calculated in comparison with controls using Student's t-test (*; p < 0.01) (**D** and **E**) Family pedigrees of Pt250 (**D**) and Pt860 (**E**). (**F**) Time-course analysis of *in vitro* amidotransferase activity of wild-type hGatCAB and mutated hGatCAB (hGatA p.G133V and p.G117A). *In vitro* amidotransferase assay was performed following the protocol provided in Methods. [^14^C]-labeled Gln and Glu deacylated from aa-tRNAs were analyzed by TLC. Positions of Gln and Glu on TLC were confirmed by [^14^C]Gln and [^14^C]Glu. Results of time-dependent amidotransferase activity are presented graphically in the lower panel.

A component of the highly conserved mitochondrial ribosome small subunit *MRPS23*[22] was mutated in Pt276, a boy with hepatic disease and combined respiratory chain complex deficiencies. In this patient, enzyme activities in complexes I and IV were decreased by 28% and 14% of the normal fibroblastic values, respectively. The patient was born to a non-consanguineous family. However, high-density oligonucleotide array analysis identified an approximately 500 kb contiguous stretch of homozygosity encompassing *MRPS23*. No other candidate gene was prioritized in our comprehensive genomic analysis. Pt276 harbored a homozygous c.119C>G (p.P40R) mutation in *MRPS23* (NM_016070) (Figs 3A and S13). Sanger sequencing identified the parents as heterozygous carriers of this mutation. A complementation assay rescued the defect in complexes I and IV (Figs 3B and S13) and restored mitochondrial 12S rRNA/16S rRNA expression (Fig 3C).

Pt250, a girl with tachypnea, hypertrophic cardiomyopathy, adrenal insufficiency, hearing loss, and combined respiratory chain complex deficiencies (I, II, III, and IV), harbored a homozygous mutation c.398G>T (p.G133V) in *QRSL1* (NM_018292) (Figs 3D and S14). Her older brother, also ill, harbored the same homozygous mutation. Sanger sequencing identified the parents as heterozygous carriers of this mutation. The high-density oligonucleotide array analysis identified a shorter 100 kb contiguous stretch of homozygosity encompassing *QRSL1*. QRSL1 (hGatA) is a glutaminase that produces ammonia, which is then transferred to misacylated Glu-charged tRNA^Gln^ to synthesize Gln-tRNA^Gln^, which interacts with PET112L (hGatB) and GATC (hGatC) to form a trimeric enzyme hGatCAB[23]. Additional screening also identified an independent patient (Pt860) harboring the compound heterozygous mutations c.350G>A (p.G117E) and c. 398G>T (p.G133V) (Fig 3E). *In vitro* reconstitution of Gln-tRNA^Gln^ formation using recombinant hGatCAB revealed strongly decreased transamidation activity in both mutant (G117E or G133V) hGatA (Fig 3F).

**Fig 4:**
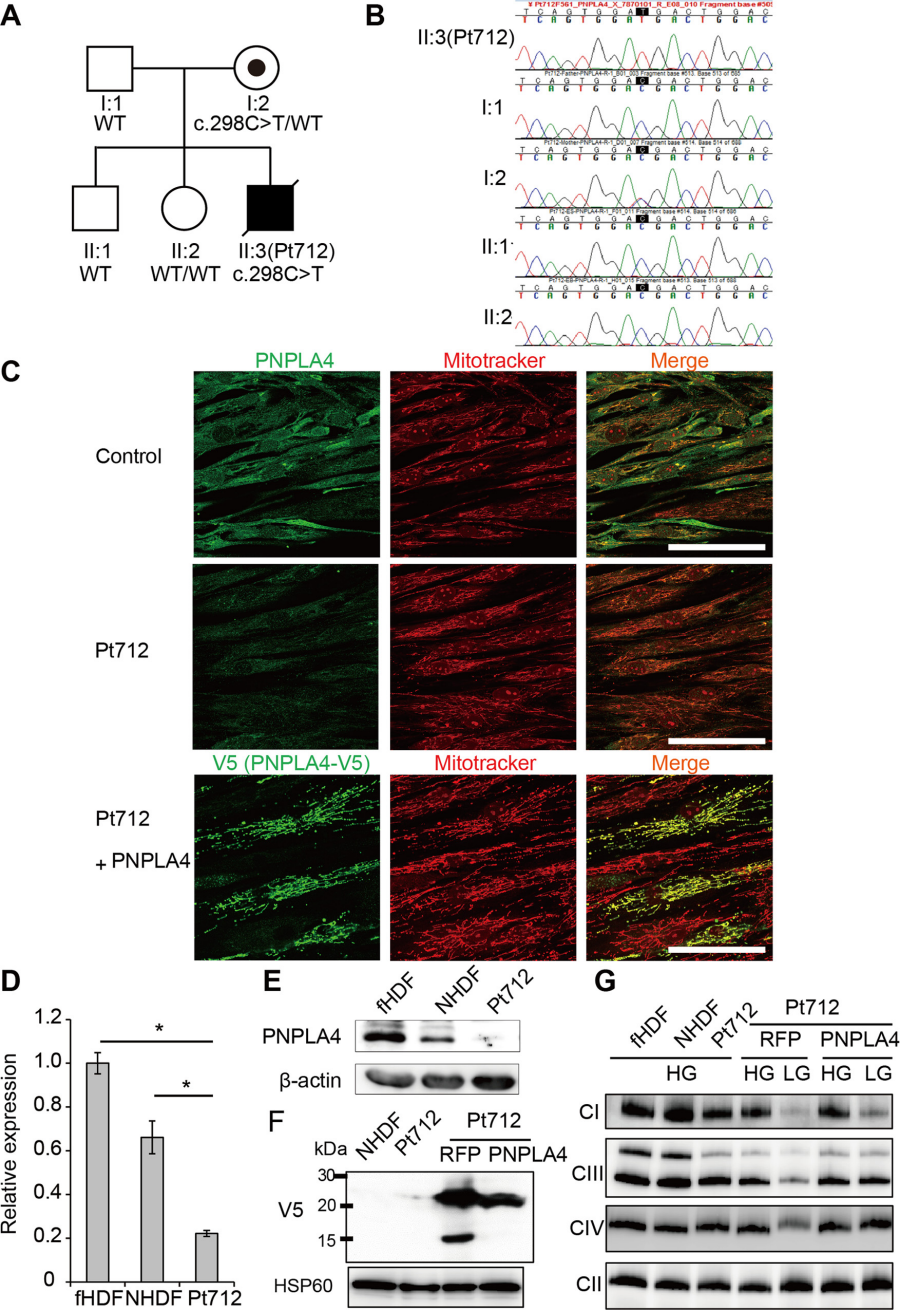
The effect of *PNPLA4* variant on mitochondrial function. (**A** and **B**) Family pedigrees of Pt712. A hemizygous variant c.298C>T (p.R100X) in *PNPLA4* (NM_001172672) was found in Pt712. The healthy elder daughter and elder brother harbored wild-type *PNPLA4*. The c.298C>T variant was inherited from the mother by Pt712. (**C**) Immunofluorescence microscopy confirmed the colocalization of PNPLA4 (green) with MitoTracker Orange CMTMRos (red). Pt712 cells showed a decrease in the endogenous PNPLA4 protein level. The lentiviral-mediated exogenous expression of PNPLA4-V5 also confirmed mitochondrial localization of PNPLA4. Scale bar, 100 μm. (**D** and **E**) *PNPLA4* mRNA and protein expression levels were determined by quantitative real-time PCR and SDS-PAGE/Western blotting. *PNPLA4* mRNA (**D**) and protein (**E**) expression levels were apparently decreased in total cell lysates of Pt712 fibroblasts. β-actin was used as a loading control. Significance relative to controls was calculated using Student’s t-test (*; p < 0.01). (**F** and **G**) Complementation assay using Pt712 fibroblasts. (**F**) mito-TurboRFP-V5 and PNPLA-V5 proteins in the mitochondria of patient cells with stable expression of *mito-TurboRFP-V5 or PNPLA4-V5* cDNA were detected by SDS-PAGE/Western blotting. HSP60 was used as a loading control. (**G**) BN-PAGE/ Western blotting under high (4.5 g/l) and low (1.0 g/l) glucose medium conditions (abbreviated to HG and LG). In low glucose medium conditions, BN-PAGE/ Western blotting analysis revealed complex IV deficiency in patient fibroblasts. Complementation with *PNPLA4* restored the complex IV assembly level in patient fibroblasts. RFP: *mito-TurboRFP-V5*, PNPLA4: *PNPLA4-V5*.

*PNPLA4* has both triacylglycerol lipase and transacylase activities. Pt712 is a boy who inherited a hemizygous nonsense variant c.559C>T (p.R187X) in *PNPLA4* (NM_001142389) from his mother (Fig 4A and 4B). The colocalization of PNPLA4 and mitochondrial markers was identified by immunofluorescence microscopic observation (Fig 4C). We confirmed PNPLA4 protein loss in the fibroblasts of this patient by qRT-PCR (Fig 4D), sodium dodecyl sulfate (SDS)-PAGE/Western blotting (Fig 4E), and immunohistochemistry (Fig 4C). We found reduced complex I, III and IV assemblies of Pt712 fibroblasts under low glucose medium conditions (Fig 4G). The expression of PNPLA4-V5 cDNA in the fibroblasts of Pt712 recovered an amount of complex III and IV assemblies under low glucose medium conditions (Fig 4F and 4G).

### Mutations in genes known to cause other monogenic diseases

In our cohort, all patients showed mitochondrial respiratory chain complex deficiencies. Intriguingly, we identified 3 cases having mutations in two genes that were previously reported to cause other monogenic diseases but not linked to canonical mitochondrial disease. These are *MECP2* and *TNNI3* (Table 2).

Pt053, a boy with complex I deficiency and seizures, diarrhea, arrhythmia, regression, respiratory failure, liver dysfunction, and hearing loss, harbored the hemizygous *de novo* mutation c.806delG (p.G269fs, rs61750241) in *MECP2* (NM_004992) (S15 Fig), a gene reported to cause Rett syndrome (MIM 312750). We also identified another boy, Pt369, who harbored the hemizygous *de novo* mutation c.17_18insG (p.A6fs) in *MECP2* (NM_001110792) (S15 Fig).

Pt827 was diagnosed with restrictive cardiomyopathy and complex I deficiency, and harbored the heterozygous *de novo* mutation c.575G>A (p.R192H, rs104894729) (S16 Fig) in *TNNI3* (NM_000363); this exact mutation was reported to cause autosomal dominant familial restrictive cardiomyopathy (MIM 115210). Electron microscopic examination also revealed abnormally shaped mitochondria with concentric cristae (S16 Fig).

### Known pathogenic copy number variations in patients with mitochondrial respiratory chain complex deficiencies

It has long been thought that patients with mitochondrial respiratory chain complex deficiencies rarely suffer chromosomal rearrangements but instead harbor mtDNA mutation, deletion, and depletion or nuclear DNA mutation. We subjected our entire cohort to a high-density oligonucleotide array to precisely evaluate the presence of any copy number variations (CNVs) of >100 kb. Intriguingly, we identified 13 patients (9.2%) harboring rare CNVs (Tables 3 and S5).

**Table 3:** Chromosomal deletions identified in patients with mitochondrial respiratory chain complex deficiencies.

ID	Clinical diagnosis	Complex type	Genetic diagnosis	Cytoband	Size (kb)	Inheritance (Parental origin)	Known genomic disorder
Pt452	CM	CIV	Firm	6q24.3-q25.1	1,675	*De novo* (Paternal)	Congenital heart defects, nonsyndromic, 2
Pt695	HD	CI	Firm	6q24.3-q25.1	1,675	*De novo* (Paternal)	Congenital heart defects, nonsyndromic, 2
Pt369	ND	CIV	pVUS	17p12	1,429	Paternal	Hereditary neuropathy with liability to pressure palsies
Pt657	MC	CIV	Firm	17p12*	1,387	N.A.	Hereditary neuropathy with liability to pressure palsies
Pt587	LIMD	CIV	Firm	22q11.21	2,691	*De novo* (Maternal)	DiGeorge syndrome/Velocardiofacial syndrome

CM, cardiomyopathy; HD, hepatic disease; LIMD, lethal infantile mitochondrial disorder; MC, mitochondrial cytopathy; ND, neurodegenerative disorder; CI, complex I deficiency; CIV, complex IV deficiency; *de novo*, confirmed *de novo* mutation by trio-based copy number analysis; *The missense mutation in C*OX10* was found in the other allele; N.A., not available.

**Fig 5:**
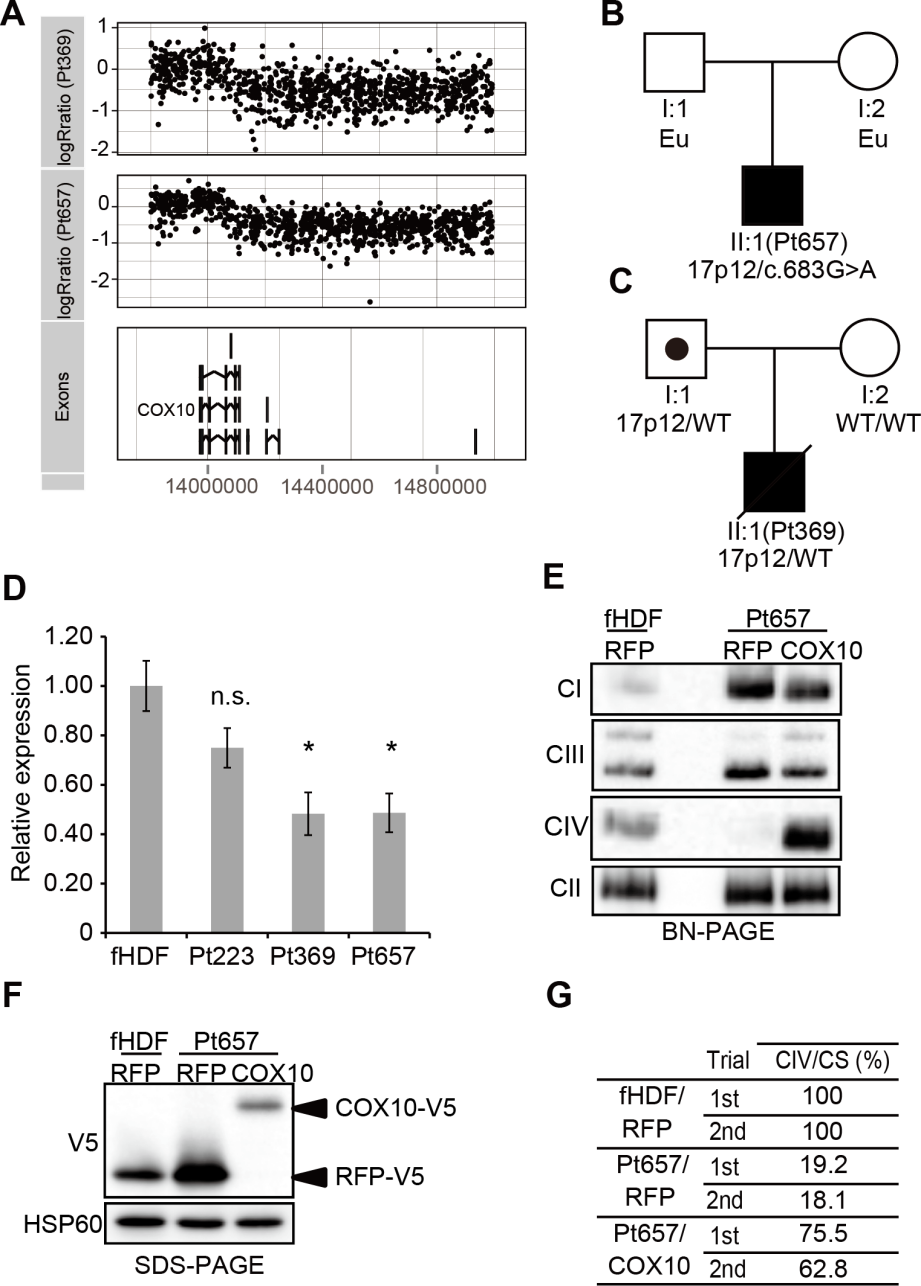
Chromosomal microdeletions contribute to mitochondrial respiratory chain complex deficiencies. (**A**) The 17p12 deletion was detected by high-density oligonucleotide arrays. This heterozygous deletion of 17p12 involved the last 2 exons of *COX10*. (**B** and **C**) Family pedigrees of Pt657 and Pt369. In Pt369, the 17p12 deletion was paternally inherited (**C**). Eu means uninformative DNA test. (**D**) Analysis of endogenous *COX10* mRNA expression relative to that in normal cells (fHDF and NHDF; Normal neonatal human dermal fibroblast); Pt223 harbored compound heterozygous non-synonymous mutations in *COX10*. The *COX10* expression in Pt369 and Pt657 was decreased by 50%. (**E**–**G**) Wild type *COX10* cDNA rescue of complex IV assembly and activity in Pt657 fibroblasts. Mitochondria were isolated from control or Pt657 fibroblasts, and mitochondrial respiratory complex assembly was analyzed by BN-PAGE and Western blotting. Wild-type COX10-V5 cDNA expression rescued complex IV assembly in Pt657 fibroblasts (**E**). Mitochondrial TurboRFP-V5 (RFP) and COX10-V5 (COX10) were detected in control and Pt657 fibroblasts (**F**). Mitochondrial respiratory chain complex activities were measured twice. Compared with TurboRFP-V5 expressing Pt657 fibroblasts, expression of wild-type COX10-V5 in Pt657 fibroblasts resulted in a significant increase in complex IV activity (**G**).

Pt369 and Pt657, 2 boys with complex IV deficiency from independent families, harbored similar deletions (1,429 and 1,387 kb) in chromosome 17p12. These 17p12 deletion disrupted the last 2 exons of *COX10* in both patients (Fig 5A). This region causes hereditary neuropathy with liability to pressure palsies (HNPP) (MIM 162500). Whole exome analysis of Pt657 revealed an additional mutation c.683G>A (p.R228H) on the remaining allele of *COX10* (Fig 5B and 5C). Notably, p.R228 is highly conserved among species. The PolyPhen2 and SIFT algorithms predicted this mutation as “probably damaging. In both patients, fibroblastic *COX10* mRNA expression was reduced (Fig 5D). The complementation study using wild-type *COX10* confirmed recovery of the complex IV deficiency in Pt657 (Fig 5E, 5F and 5G). Taken together, we concluded that Pt657 is a primary mitochondrial disorders. In Pt369, we concluded that *de novo* frameshift insertion mutation in *MECP2* as a primary causative based on phenotype information, and classified 17p12 as pVUS.

We identified *de novo* 6q24.3-q25.1 deletions (S17 Fig) in Pt452 and Pt695, unrelated patients who harbored congenital heart defects. This region has been associated with the chromosome 6q24-q25 deletion syndrome (MIM 612863) and congenital heart defects[24].

Pt587, a boy diagnosed with LIMD and complex IV deficiency, harbored a deletion in 22q11.21. This deletion, which has been linked to DiGeorge syndrome (DGS, MIM 188400) and velo-cardio-facial syndrome (VCFS, MIM 192430), was confirmed as a *de novo* mutation in this patient (S17 Fig).

## Discussion

We performed comprehensive genomic analyses, including whole mtDNA and exome sequence analyses using high-throughput sequencing and CNV screening using high-density oligonucleotide arrays, for 142 patients with childhood-onset mitochondrial respiratory chain complex deficiencies. We ultimately identified 41 mutations, of which 37 were novel, in 20 genes that were previously reported to cause OXPHOS disease and 3 novel mitochondria-related genes (*MRPS23*, *QRSL1*, *and PNPLA4*) as causative genes of mitochondrial respiratory chain complex deficiencies. We also found 9 previously confirmed mtDNA mutations and 1 large mtDNA deletion. We further identified 2 genes known to cause monogenic diseases (*MECP2*, and *TNNI3*) and 3 chromosomal aberration regions (17p12, 6q24.3-q25.1, and 22q11.21) in our cohort. Collectively, this study defined firm genetic diagnoses in 49 of the 142 patients (34.5%). While the overall diagnostic rates for major and minor subgroups were similar (33.9% in the major subgroup and 36.4% in the minor subgroup), 35 out of 49 genetically diagnosed patients showed biochemical defects in their fibroblasts (71.4%), indicating a much higher genetic diagnostic yield in patients with such cellular defects. This is the first report to comprehensively assess patients diagnosed clinically and biochemically.

MRPS23 is a component of the small mitochondrial ribosome subunit (28S ribosome). Mutations in *MRPS16*[25] and *MRPS22*[26] cause mitochondrial respiratory chain complex deficiencies because of reductions of 12S rRNA, a 28S ribosome component. One patient exhibited a reduced 12S rRNA/16S rRNA ratio that was restored in a complementation study. This was the first case of *MRPS23*-induced mitochondrial respiratory chain complex deficiencies.

QRSL1 (GatA) is involved in Gln-tRNA^Gln^ formation. No mitochondrial glutaminyl-tRNA synthetase (GlnRS) has been identified in mammals; therefore, Gln-tRNA^Gln^ synthesis was proven to occur via an indirect pathway[23]. In particular, mt tRNA^Gln^ is first misaminoacylated by mt glutamyl-tRNA synthetase (GluRS) to form Glu-tRNA^Gln^, followed by transamidation to form Gln-tRNA^Gln^. This transamidation is processed by a human homolog of the Glu-tRNA^Gln^ amidotransferase hGatCAB heterotrimer. We clearly showed that mutations in QRSL1 (GatA), a component of hGatCAB, observed in our patients were associated with severe transamidation activity defects.

*PNPLA4* encodes a calcium-independent phospholipase A2η (iPLA2η) that acts as an acylglycerol and retinol transacylase, triglyceride hydrolase. PNPLA4 has never been reported to associate with the mitochondria. Nine patatin-like phospholipase domain-containing proteins (PNPLA1–9) are encoded in the human genome. iPLA2γ (PNPLA8) is known to be involved in cardiolipin biosynthesis and mitochondrial respiration[27,28]. Recently, mutations in human *PNPLA8* identified in a young girl with a suspected mitochondrial myopathy[29]. She presented with progressive muscle weakness, hypotonia, seizures, poor weight gain, and lactic acidosis. A deficiency in iPLA2β (PNPLA9) was previously shown to cause abnormal phospholipid metabolism and mitochondrial defects in mice[30]. Here we demonstrated the mitochondrial localization of iPLA2η using immunohistochemistry and restored of the amount of complex IV in Pt712 fibroblast cells via the exogenous expression of wild-type *PNPLA4*. We assume that *PNPLA4* is also required for mitochondrial phospholipid metabolism and respiratory chain function.

Although all patients were diagnosed with mitochondrial respiratory chain complex deficiencies, we identified 2 disease-causing genes and 2 pathogenic CNVs known to cause other genetic disorders in our cohort. These included *MECP2*, *TNNI3*, 6q24.3-q25.1 deletion, and 22q11.21 deletion. Because all of these patients had complex II activities within the normal range (percentage of protein and citrate synthase ratio), we concluded that their defects were not artefactual[31,32]. Because these genes and loci are not directly linked to the respiratory chain complex, we consider the mitochondrial respiratory chain complex deficiencies are caused by secondary. Pt053, Pt369, and Pt827 were classified as having major ETC reductions in affected tissues, whereas Pt452, Pt695, and Pt587, harbored deletions are all classified as minor. The fact these heterozygous deletions are all classified as minor suggests that the mitochondrial defects in these patients might be caused indirectly through haploinsufficiency.

Because Pt053 and Pt369 harbored *MECP2* mutations known to cause Rett syndrome, we re-evaluated the phenotypes of both patients and found phenotypes that overlapped with Rett syndrome characteristics (seizures, microcephaly, cerebral atrophy, and hearing loss). Previous studies also reported mitochondrial dysfunction in Rett syndrome[33,34]. Although Pt827 was enrolled with a diagnosis of mitochondrial disease, after comprehensive genomic analyses, the clinical diagnosis was changed to cardiomyopathy, familial restrictive (OMIM: 115210) caused by a mutation in *TNNI3*. Jia et al reported a link between Tnni3 and mitochondrial dysfunction using knockout mice [35].

Two independent patients from our cohort showed 6q24.3-q25.1 deletions. Pt452 exhibited a phenotype similar to that of cases reported cases in OMIM (MIM 612863). Pt695 presented with respiratory distress and a congenital heart defect. We classified these patients as having chromosome 6q24-q25 deletion syndrome. The enrichment of this deletion supports the suggested link with mitochondrial dysfunction. Pt587 was difficult to diagnose based on clinical information, because he did not have the facial anomalies and cleft palate characteristic of DGS/VCFS. The 22q11.21 deletion includes some mitochondria-related genes (*PRODH*, *SLC25A1*, *MRPL40*, *TXNRD2*, *COMT*, *TANGO2*, *ZDHHC8*, and *AIFM3*), suggesting a link between this deletion and mitochondrial dysfunction. The inclusion of a patient with features of DGS/VCFS and complex I deficiency in a study by Calvo et al[36] also indicates a link with mitochondrial dysfunction.

With these in mind, we should be mindful that some patients with mitochondrial respiratory chain complex defects will have mutations in genes apparently unrelated to mitochondrial functions.

Previous reports of mitochondrial disorders can be classified as either target resequence studies[37,38,7] or whole exome approaches[39,40,41]. When comparing target resequencing groups, our approaches are advantageous for the identification of mutations in other disease-causing genes, and the detection of chromosomal aberrations. WES groups[40,41] and our group detected pathogenic mutations in genes not linked to mitochondrial disorders. When comparing WES groups, our approaches are advantageous in terms of mtDNA sequencing and chromosomal aberration analysis. Our analysis could detect mtDNA heteroplasmy using long-range PCR-based sequencing and also revealed established pathogenic chromosomal deletions. Accordingly, we identified a composite combination of *COX10* SNV and 17p12 deletion (Pt657). Previous WES and target exome reports achieved molecular diagnoses in 20%–60% of their cohorts. A precise comparison of overall diagnostic rates with previous studies is difficult, given the existence of several biases that affect the diagnostic rate, including prior mtDNA/nDNA genetic screening, population characteristics, phenotyping accuracy, and study design. In particular, the reports by Taylor et al[40] described a high rate of diagnosis (approximately 60%) in their cohort, although their patient group appeared to be enriched by consanguineous families (12 of 28 diagnosed cases). The report by Wortmann et al[41] described a rate of diagnosis (38%) similar to ours. In our study, we emphasized functional analyses to conclude disease causality against pVUS and attempted to present molecular evidence of pathogenicity;in contrast, some previous studies lacked sufficient molecular evidence of pathogenicity. We designated the variants without any molecular evidence of pathogenicity as pVUS, even when the gene had been reported as a causal gene for mitochondrial disorders. We consider molecular evidence to be indispensable for a conclusive firm genetic diagnosis.

We found that approximately 28.2% of patients lacked any prioritized variants. We likely missed pathogenic mutations in these unresolved cases for the following reasons, as discussed in a report by Calvo et al[6]: first, we may have missed pathogenic mutations because of a lack of sensitivity from low sequence coverage. Second, pathogenic mutations may be located in uncovered genomic regions (e.g., uncovered exons, introns, or regulatory regions not targeted by whole exome platforms). Third, our filtering strategy may have filtered true pathogenic mutations, although some were recovered by manual curation. Fourth, the hereditary assumption may be wrong. More dominant-acting cases may exist. Digenic/polygenic inheritance may also exist beyond our expectation.

In conclusion, for suspected mitochondrial disorders, comprehensive analyses such as those in this study are worthwhile. We expanded the clinical disease spectrum and revealed the genetic landscape of this disorder.

## Methods

### Patient information

In total, 142 patients with childhood-onset and enzymatically diagnosed mitochondrial respiratory chain complex deficiencies were enrolled in this study. Informed consent was obtained from the patients and their families before participation in the study. Patients with suspected mitochondrial respiratory chain complex deficiency were referred to the Saitama Medical University Hospital and Chiba Children’s Hospital in Japan from 2007 to 2013. The inclusion criterion was a biochemical diagnosis of mitochondrial respiratory chain complex activity in a clinically affected tissue (skeletal muscle, liver, or heart) or fibroblasts in patients younger than at the age of 15 years. Patients with known nuclear or mtDNA mutations at the time of recruitment were excluded. The 142 included patients had not received a prior molecular diagnosis, despite varying degrees of exposure to genetic testing. This cohort included 3 non-Japanese cases: Pt346 (father, American; mother, Japanese), Pt298 (Brazilian), and Pt223 (Vietnamese). Enzyme activity[42] was measured on the basis of spectrophotometric enzyme assays using fibroblasts from patient’s skin or biopsy specimens from diseased organs of patients with clinically suspected mitochondrial respiratory chain disorders[43]. All enrolled patients in this study had biochemical mitochondrial respiratory chain complex deficiencies; the enzymatic diagnoses are shown in S1A Fig. In brief, complex I deficiency was most common (61 patients, 43.0%), followed by (in decreasing order of prevalence) combined respiratory chain complex deficiencies (46 patients, 32.4%), complex IV deficiency (27 patients, 19.0%), MTDPS (5 patients, 3.5%), and complex III deficiency (3 patients, 2.1%); no patients exhibited complex II deficiency. Diagnoses of mitochondrial respiratory chain complex deficiencies were assessed as “major” or “minor” on the basis of biochemical complex activity. Based on the Bernier criteria, severity was defined as major (<20% in a tissue, <30% in a fibroblast cell line, or <30% in ≥2 tissues) or minor (<30% in a tissue, <40% in a fibroblast cell line, or <40% in ≥2 tissues) in accordance with the residual mean citrate synthase or complex II activities relative to those of normal controls (S1B Fig). The distribution of age of onset of these patients was as follows: 45.7% (65 patients) before 1 month, 19.7%(28 patients) within 1–6 months, 19.7%(28 patients) within 6–24 months, 12,7% (18 patients) within 2–10 years, and 2.1% (3 patients) within 10–15 years (S1C Fig). The clinical diagnoses of 142 patients are also shown in S1D Fig. The most common diagnosis was mitochondrial cytopathy (27 patients, 19.0%), followed by Leigh’s disease (25 patients, 17.6%), LIMD (23 patients, 16.2%), sudden unexpected death (17 patients, 12.0%), non-lethal infantile mitochondrial disorder (NLIMD) (16 patients, 11.3%), cardiomyopathy (11 patients, 7.7%), hepatic disease (11 patients, 7.7%), enteropathy (6 patients, 4.2%), neurodegenerative disorder (4 patients, 2.8%), and short stature (2 patients, 1.4%). The male:female ratio was 76:66. There were no consanguineous relationships among our cohort. Detailed clinical characteristics are described in S1 Table.

### DNA and RNA extraction and cDNA preparation

DNA was isolated from cultured fibroblast cells using the QIAamp DNA Blood mini Kit (QIAGEN). Blood genomic DNA was isolated by phenol–chloroform extraction according to the standard protocol. Total RNAs were purified from HEK293FT cells, fibroblast cells using the SV Total RNA Isolation System (Promega). cDNAs were synthesized from total RNAs using ReverTra Ace (Toyobo). Total RNA was extracted from flies using TRIzol reagent (Invitrogen), and RNA was reverse transcribed by SuperScript VILO transcriptase (Invitrogen).

### mtDNA sequencing and alignment and variant prioritization

To avoid the contamination of mitochondrial-origin nuclear genome sequences [44] (NUMTs), a long-range mtDNA polymerase chain reaction (PCR) method was used in this study. DNA were extracted from patients skin fibroblast cells. These DNAs were checked for large-scale mtDNA rearrangements and subjected to large mtDNA deletion mapping using long-range PCR with amplicon 1 (rCRS 619–8988) and amplicon 2 (rCRS 8749–895) primers; 5′-GACGGGCTCACATCACCCCATAA-3′ and 5′-GCGTACGGCCAGGGCTATTGGT-3′ for amplicon 1, and 5′-GCCACAACTAACCTCCTCGGGCTCCT-3′ and 5′-GGTGGCTGGCACGAAATTGACC-3′ for amplicon 2.

Indexed PCR fragment libraries were prepared from patient mtDNA using the Nextera XT DNA Sample Prep Kit (Illumina) according to the manufacturer’s protocol. Sequencing library concentrations were estimated using a library quantification kit (Kapa Biosystems). Sequencing was performed with 150-bp paired-end reads on MiSeq (Illumina). Read alignments to the 1000 Genomes Project phase II reference genome (hs37d5.fa), which contains rCRS sequences, were performed with the Burrows–Wheeler Aligner[45] (BWA, version 0.7.0). PCR duplicate reads were removed using Picard (version 1.89); non-mappable reads were removed using SAMtools[46] (version 0.1.19). After filtering out those reads, we applied the Genome Analysis Toolkit[47] (GATK version 2.4-9-nightly-2013-04-12-g3fc5478) base quality score recalibration and performed SNP and INDEL discovery (UnifiedGenotyper). Confirmed pathogenic mutations and reported variants in MITOMAP and mtDNA deletions detected through reference-based alignment with BWA mapping were prioritized (S4 Table).

### mtDNA *de novo* assembly

*De novo* mtDNA sequence assembly was performed using SPAdes (version 3.0.0)[48] with iterations over values of 3 kmer sizes (k = 75, 95, and 115). Each assembly was aligned to the mitochondrial genome sequence of hs37d5.fa using BLASTN (version 2.2.29+) with default settings and was manually inspected to identify aberrations (deletions, duplications, and rearrangements).

### Validation of large mtDNA deletion

A large mtDNA deletion in Pt334 was validated using long-range PCR with primers 5′-GCCACAACTAACCTCCTCGGGCTCCT-3′ and 5′-GGTGGCTGGCACGAAATTGACC-3′. The mtDNA was also sequenced (using primers 5′-ACTACCACTGACATGACTTTCCAA-3′ and 5′-TGTTGTTTGGATATATGGAGGATG-3′ for amplification and 5′-CTTATCCAGTGAACCACTATCACG-3′ for sequencing) closer to the breakpoint as described above.

### Quantitative PCR for MTDPS diagnosis

Quantitative PCR[49] was used to determine whether mtDNA depletion was present in patients with decreased activity levels for multiple respiratory chain enzymes (mtDNA gene *MT-ND1* was compared against a nuclear gene [*CFTR* exon 24]). A diagnosis of MTDPS was made when the relative copy number ratio of mtDNA to nuclear DNA was less than 35% of that in healthy control tissues in 4 independent experiments.

### Quantitative reverse transcription PCR

Quantitative reverse transcription PCR (qRT-PCR) was performed for the analysis of mRNA (*NDUFB11*, *TTC37*, and *PNPLA4*) and mitochondrial rRNA expression[50]. Primers were designed with the Primer3 software[51]. Primer sequences used in the qRT-PCR analysis are listed in S6 Table. qRT-PCR of cDNA extracted from human cells was performed using SYBR Premix Ex Taq (Takara), Power SYBR Green PCR Master Mix (Life technologies), and Mx3000P (Agilent Technologies). The relative mRNA concentration was normalized to the average of two housekeeping genes (β-actin and GAPDH). qRT-PCR of cDNA extracted from flies was performed using SYBR Premix Ex Taq II (Takara) and Chromo 4 Four-Color Real-Time System (Bio-Rad). Results were normalized to the *rp49* mRNA level.

### Whole exome sequencing and variant calling pipeline

Indexed genomic DNA (gDNA) libraries were prepared from patient gDNA, and exomes were captured using TruSeq (Illumina), SeqCap EZ (Roche AG, Basel, Switzerland), and SureSelect (Agilent Technologies) exome enrichment kits according to the manufacturers’ protocols. Sequencing library concentrations were estimated using a library quantification kit (Kapa Biosystems). Sequencing was performed using 100-bp paired-end reads on a HiSeq2000 or GAIIx (Illumina). The precise exome platforms used in this study are listed in S2 Table. The raw sequence read data passed the quality checks in FASTQC (see URLs). Read trimming via base quality was performed using Trimmomatic[52]. Read alignment was performed with BWA, the hs37d5 reference genome, Picard, and SAMtools as described above. GATK was also used for insertion and deletion realignment, quality recalibration, and variant calling. Detected variants were annotated using both ANNOVAR (version 2013Feb21)[53] and custom ruby scripts. Prediction scores were obtained from dbNSFP[54].

### Whole exome variant prioritization

Variants that passed quality control were prioritized according to the following strategies (S2 Fig). We only retained variants predicted to modify protein function; these included nonsense, splice site, coding indel, or missense variants. We removed variants with minor allele frequencies (MAFs) of >1.0% for dbSNP 137 without known medical impact (allele frequencies were extracted from common_no_known_medical_impact_20130808.vcf.gz), >0.1% for ESP6500 (provided by ANNOVAR program) database, >1.0% for 1KG (these data are based on a phase 1 release v3 called from the 20101123 alignment and provided by ANNOVAR), >0.1% for the Exome Aggregation Consortium (ExAC, accessed on December 2014), and >0.4% in HGVD (contains genetic variations determined by exome sequencing of 1,208 individuals in Japan; see URLs). Variants that were too common among cases (≥10 alleles) were also excluded. Careful inspection of the reads using the Integrative Genomics Viewer[55,56] and NextCODE clinical sequence analyzer (see URLs) excluded doubtful genes from prioritized candidate genes when 2 sequence variants were present in the same read (or read-pair). Variants that appeared to be mapping artifacts (called by suspicious reads or end positions of NGS reads) were also excluded through a manual inspection of NGS reads. Variants located within segmental duplication regions were excluded. In addition to these filters, Sorting Intolerant From Tolerant (SIFT) scores > 0.15 and Genomic Evolutionary Rate Profiling (GERP) scores < 2.5 were used for further prioritization. We also excluded variants inconsistent with a recessive mode of inheritance. Two (or more) variants on a single haplotype as identified by Sanger sequencing were also excluded. Finally, we filtered remaining genes based on MAF and genotype information in the 1,070 whole-genome reference panel database (1KJPN) constructed in the Tohoku Medical Megabank Project in Japan (http://ijgvd.megabank.tohoku.ac.jp/). The details of the project and analysis are described in the 1KJPN literature[57]. To recover true mutations that were filtered out using current pipeline applying stringent conditions, we also applied this pipeline without a segmental duplication filter, SIFT filter, or GERP filter, followed by focusing on mitochondria-related genes.

### Prioritized gene enrichment analysis

Enrichment analysis was conducted to evaluate our exome pipeline and included 128 cases and 175 ethnically matched healthy controls whose sequence reads exceeded 50 million; these were adjusted to 50 million reads per individual. We used a simplified exome analysis pipeline that did not include a manual inspection step, Sanger sequencing validation step, and 1KJPN filtering step. We also omitted the HGVD filtering step because these control data were included in the HGVD samples. The other steps were the same as those described above for the exome analysis pipeline. After processing the controls and cases, we calculated the percentage of individuals harboring prioritized genes. Comparisons of the percentages of controls and cases on the basis of known disease genes and mitochondria-related genes are shown in S3A Fig. To consider the background rate of this simplified pipeline, we also evaluated enrichment using randomly selected 908 genes with no strong mitochondrial relationships in their annotations. The gene set was generated 1000 times via random selection from all genes after excluding those known to cause OXPHOS disease and those listed in MitoCarta[18]. The results plus standard deviations are shown in S3B Fig.

### Sequence validation and haplotype phasing

Prioritized variants were independently validated by Sanger sequencing. PCR products were either directly sequenced using GENEWIZ, ABI 3130XL, and BigDye v3.1 Terminators (Applied Biosystems) per the manufacturer’s protocols or sequenced after gel purification using the MinElute Gel Extraction Kit (QIAGEN). Sequencing primers are listed in S7 Table. All compound heterozygous variants described in the main text were confirmed on different alleles (phased) using sequenced, cloned gDNA or cDNA derived from the patients’ fibroblasts. Patients’ familial DNA was also sequenced for haplotype phasing when available. All information about the experimentally confirmed localization of compound variants within a separate allele is presented in S3 Table.

### Multiple alignment with ClustalW

Amino acid sequences of orthologous genes were downloaded from the HomoloGene database (see URLs). Amino acid sequence alignments were constructed with the ClustalW2 program[58].

### Cell culture and cDNA rescue assay

Cells were cultured at 37°C and 5% CO_2_ in Dulbecco's modified Eagle’s medium (DMEM 4.5 g/l glucose or 1.0 g/l glucose; Nacalai tasque) supplemented with 10%–20% fetal bovine serum. Normal neonatal human dermal fibroblasts (NHDFs; Takara) and normal fetal human dermal fibroblasts (fHDFs; Toyobo) were used as control fibroblast cells. Open reading frames (ORFs) of candidate genes (*ACAD9*, *BOLA3*, *COX10*, *KARS*, *MRPS23*, *NDUFA10*, *NDUFAF6*, *PNPLA4*, and *TUFM*) were PCR amplified from cDNA. Primer sequences used for cDNA cloning are listed in S8 Table. ORFs and pTurboRFP-mito (TurboRFP fused to a mitochondrial targeting sequence derived from the subunit VIII of human cytochrome C oxidase; Evrogen) were cloned into the CS-CA-MCS lentiviral vector with a C-terminal V5 tag, CAG promoter for mammalian cell expression, and blasticidin resistance using the In-Fusion HD Cloning Kit (Clontech Laboratories, Inc.). Following this, 2 × 10^6^ HEK293FT cells were seeded in 6-cm plates and co-transfected with ViraPower Packaging vectors (pLP1, pLP2, pLP/VSVG; Invitrogen) and a pCA-CS-ORF(candidate gene)-blast vector. Transfection was performed using Lipofectamine 2000 (Invitrogen). Transfection medium was replaced with fresh medium 24 h after transfection. Supernatant containing the viral particles was collected 48 h after transfection and filtered through a 0.45 μm filter. Patients’ skin fibroblasts were infected with the viral supernatant and 5 μg/ml polybrene (Sigma) for 24–48 h. After 5–7 days, selection was initiated with 1–2 μg/ml blasticidin. After 1–3 months of selection, mitochondria were harvested from the cells for enzyme assays or BN-PAGE.

### Respiratory chain enzyme analysis

To prepare enriched mitochondria, cell pellets were resuspended in ice-cold MegaFb Buffer (250 mM sucrose, 2 mM HEPES, 0.1 mM EGTA, pH 7.4) and homogenized with 20 strokes. The homogenates were centrifuged for 10 min at 600 *g*. Supernatants were centrifuged for an additional 10 min at 14,400 *g*. Pellets were resuspended in 400 μl MegaFb buffer, and 200 μl aliquots were frozen and thawed 3 times for complex II + III and complex III assays and protein estimation. The remaining samples were resuspended in hypotonic buffer (25 mM potassium phosphate, pH 7.2, 5 mM MgCl_2_) for complex I, II, and IV, citrate synthase, and protein concentration assays and centrifuged for 10 min at 14,400 *g*. Pellets were resuspended in Hypotonic Buffer and subjected to 3 freeze–thaw cycles. These samples were stored at −80°C prior to enzyme assays. Respiratory chain enzyme activities were measured using cary300 (Agilent Technologies) as described previously[42]. Complex I, II, II + III, III, and IV activities were expressed as percentages of citrate synthase activity.

### SDS-PAGE and BN-PAGE analyses

To isolate mitochondria, cell pellets were suspended in mitochondria isolation buffer A (220 mM mannitol, 20 mM HEPES, 70 mM sucrose, 1 mM EDTA, pH 7.4, 2 mg/ml bovine serum albumin, 1× protease inhibitor cocktail) and homogenized with 20 strokes on ice. Homogenates were separated into cytosolic and nuclear fractions after centrifugation at 700 *g* for 5 min at 4°C. The supernatants were centrifuged at 10,000 *g* for 10 min at 4°C. Mitochondrial pellets were rinsed twice with mitochondria isolation buffer B (220 mM mannitol, 20 mM HEPES, 70 mM sucrose, 1 mM EDTA, pH 7.4, 1× protease inhibitor cocktail). Mitochondria were isolated from adult flies as described previously[59]. Fifty flies were homogenized in 1 ml of chilled mitochondrial isolation medium (MIM; 250 mM sucrose, 10 mM Tris pH 7.4, 0.15 mM MgCl_2_). The samples were centrifuged twice for 5 min at 1,000 *g* at 4°C to remove debris. The supernatant was centrifuged again for 5 min at 13,000 *g* at 4°C.Mitochondrial protein levels were determined using a bicinchoninic acid (BCA) assay. For SDS-PAGE analyses, enriched mitochondria and cell pellets were solubilized in M-PER Mammalian Protein Extraction Reagent (Thermo Fisher Scientific) and denatured for 30 min at 37°C. Prepared samples were separated by electrophoresis on 8%, 10%, and 15% SDS-PAGE gels, depending on the size of the detected protein. For BN-PAGE analyses, The NativePAGE Novex Bis-Tris Gel System (Life Technologies) was used according to the manufacturer’s protocol. Mitochondrial fractions were solubilized in NativePAGE sample buffer containing 0.5% Triton-X100 and separated on 4%–16% NativePAGE gels. The BN-PAGE analyses of *Drosophila* were performed as previously described[60]. Immunoblot analysis was performed as described previously[61]. Anti-NDUFA9 (Complex I), anti-70 kDa Fp Subunit (Complex II), anti-core 1 (Complex III), anti-subunit 1 (Complex IV), and anti-V5 antibodies were purchased from Life Technologies. Anti-Lamin A/C antibody was purchased from BD biosciences. Anti-HSP60, anti-NDUFA10, anti-ACAD9, and anti-COX10 antibodies were purchased from Abcam, and anti-NDUFB11 antibody was purchased from Santa Cruz Biotechnology. Anti-TTC37 antibody was purchased from ProteinTech. Anti-PNPLA4 (GS2) was purchased from GeneTex. Anti-tafazzin and anti-α/β-tubulin antibodies were purchased from Cell Signaling Technology. Anti-β-actin antibody was purchased from Sigma. Anti-MECP2 antibodies were purchased from Acris Antibodies and Merck Millipore.

### Localization studies of the PNPLA4 protein

Normal human dermal fibroblast cells and patient cells were seeded in a 35-mm glass-bottom dish. Mitochondria were stained with 500 nM MitoTracker Orange CMXRos (Molecular Probes) for 30 min in DMEM containing 10% fetal bovine serum. Cells were fixed with 4% paraformaldehyde for 20 min and permeabilized by incubation in 0.2% Triton X-100. After blocking with 3% bovine serum albumin, fluorescent staining was performed with rabbit anti-PNPLA4 antibody (GeneTex) or mouse anti-V5 antibody (Life Technologies) and secondary Alexa Fluor 488 antibody (Molecular Probes) or secondary FITC antibody (Sigma). Cells were visualized with a Leica TCS SP8 confocal microscope.

### siRNA knockdown

For siRNA transfection, Lipofectamine RNAiMAX (Invitrogen) and 120 pmol of siRNA were prepared according to the manufacturer’s instructions and directly added to a 10 cm culture dish of NHDF fibroblasts. Mitochondria were isolated after 6 days, and the assembly levels of respiratory chain complexes were analyzed using BN-PAGE and Western blotting. The Stealth RNAi siRNA (Life Technologies) sequences used for *NDUFB11* knockdown are as follows: HSS147694 (#94), ACC CAG ACU CCC AUG GUU AUG ACA A; HSS147695 (#95), UCC AAG AGC GUG GGA UGG GAU GAA A; HSS147696 (#96), CCU CUU CUC AGA GCA CCU AAU UAA A. Stealth RNAi siRNA Negative Control, Med GC (cat no. 12935–300) was used as the negative control. The Silencer Select RNAi siRNA (Life Technologies) used for *MECP2* knockdown are as follows: s8644 (#44), s8645 (#45), s8646 (#46). Silencer Select RNAi siRNA Negative Control, No.2 (#2) (cat no. 4390847) was used as the negative control.

### Fly strains and culture conditions

Flies were reared at 25°C in a standard glucose yeast agar medium containing propionic acid and *n*-butyl *p*-hydroxybenzoate as mold inhibitors. *arm-Gal4* was obtained from the Bloomington *Drosophila* Stock Center. *UAS-dndufb11 (NP15*.*6)-IR* (5717) was obtained from the Vienna *Drosophila* RNAi Center. *UAS-GFP-IR (GFP-IR-2)* was obtained from the National Institute of Genetics Fly Stock Center.

### Lifespan measurement

Newly eclosed flies were housed in a glass vial containing the standard glucose yeast medium and were transferred to fresh media every 2 days; the numbers of dead flies were counted at the time of transfer. At least 100 flies per genotype were used for experiments.

### Behavioral analysis

Eight flies were placed in an 8-lane cell vial (1 cell; H 7 mm × W 8 mm × D 70 mm) and bumped to the bottom. Pictures were taken at 5 s after bumping and used to measure the distance climbed by each individual. For each sample, the average climbing activity of 10 trials was determined.

### Measurement of CO_2_ production

CO_2_ production was measured as described previously[62]. In brief, 10 adult flies were placed in a 1-ml plastic syringe that contained a small amount of CO_2_-absorbent material (Soda lime), which was connected to a 200-μl glass disposable micropipette. A small amount of black ink was placed at the end of the micropipette as an indicator of CO_2_ production. The apparatus was kept on a flat surface at 25°C, and measurement was initiated after 10 min. The amount of CO_2_ produced by the flies was calculated according to changes in the air volume during 1 h of measurement. Assays were performed at least 3 times per genotype.

### Measurement of lactate and pyruvate

Lactate and pyruvate measurements were performed as described previously[63].

### Preparation of hGatCA complex bearing pathogenic mutation

The cDNAs for hGatA with C-terminal SBP-HA-tag and hGatC with C-terminal HA-tag were cloned into pENTR/D-TOPO (Invitrogen). Each of the pathogenic point mutations (G117E and G133V) was introduced into the hGatA gene in the entry clone by site-directed mutagenesis using PrimeSTAR HS DNA polymerase (Takara) with primers 5′-GATCAGGGAGCTCTACTAATGGAAAAAACAAATTTAGA-3′ and 5′-TCATCTAAATTTGTTTTTTCCATTAGTAGAGCTCCCTGATC-3′ for G117E, and 5′-GATCTGGGAGCACAGATGTTGTATTTGGACCAGTTAAAAAC-3′ and 5′-GTTTTTAACTGGTCCAAATACAACATCTGTGCTCCCAGATC-3′ for G133V. The cDNAs for hGatA (WT, G117E or G133V) and hGatC were transferred from each entry clone to pHAGE to generate the expression vector[64] by LR reaction (Invitrogen).

HEK293T cells were co-transfected with lentiviral vectors (TAT, VSVG, RRE, or REV), pHAGE-hGatA (WT, G117E or G133V) and pHAGE-hGatC. The transformants were cultured at 37°C for 3 days. Cells were harvested and suspended in lysis buffer [50 mM HEPES-KOH (pH 7.5), 200 mM KCl, 1 mM PMSF, 0.1% TritonX-100, 1 mM DTT, 2.5 mM MgCl_2_] containing complete protease inhibitor cocktail (Roche) and were disrupted by sonication at 0°C. The hGatCA complex in the cell lysate was captured with streptavidin-Sepharose beads (GE Healthcare) and was eluted from the beads with 4 mM of biotin according to the manufacturer’s instructions. The eluted hGatCA complex was subjected to SDS-PAGE, stained by SyproRuby, and quantified with a FLA-7000 imaging analyzer (Fujifilm) with BSA as a standard. Recombinant hGatB was expressed in *Escherichia coli* and was purified as described previously[23].

### [^14^C] glutamyl-tRNA^Gln^ preparation

As human mt GluRS strictly recognizes the post-transcriptional modification at the anticodon first position (position 34) of human mt tRNA^Gln^ for glutamylation[23], *in vitro*-transcribed human mt tRNA^Gln^ cannot be aminoacylated by human mt GluRS. However, *Thermotoga matritima* nondiscriminating GluRS can efficiently glutamylate tRNA^Gln^ bearing unmodified C at position 34[65]. We accordingly prepared *in vitro*-transcribed human mt tRNA^Gln^ with C34 for glutamylation by *T*. *maritima* GluRS. Human mt tRNA^Gln^ with C34 was transcribed *in vitro* by T7 RNA polymerase from the template DNA PCR-amplified with the synthetic DNAs 5′-GCTAATACGACTCACTATATAGGATGGGGTGTGATAGGTGGCACGGAG-3′, 5′-ATAGGTGGCACGGAGAATTCTGGATTCTCAGGGATGGGTTCGAT-3′, and 5′-TGGCTAGGACTATGAGAATCGAACCCATCCCTGA-3′, as described previously[66,67]. The aminoacylation reaction was performed at 37°C for 30 min in a mixture containing 50 mM HEPES-KOH(pH 7.5), 20 mM KCl, 10mM MgCl_2_, 2 mM ATP, 1 mM DTT, 1 mM spermidine, 20 μM [^14^C]L-glutamine(9.36 GBq/mmol), 0.02 A_260_ unit of tRNA transcript, and 1.88 μM *T*. *maritima* GluRS. The [^14^C]Glu-tRNA^Gln^ was extracted by phenol–chloroform treatment under acidic conditions followed by ethanol precipitation. Residual ATP in the reaction was removed using a Nap5 gel filtration column (GE Healthcare). A small part of the mixture was spotted onto Whatman 3MM filter discs, followed by washing with 5% trichloroacetic acid, and the radioactivity was measured by liquid scintillation counting.

### Glutaminyl-tRNA^Gln^ formation by hGatCAB *in vitro*

*In vitro* reconstitution of Gln-tRNA^Gln^ formation by hGatCAB was performed as described previously[23]. The reaction was performed at 37°C in a mixture of 100 mM HEPES-KOH (pH 7.5), 30 mM KCl, 12 mM MgCl_2_, and 2.5 mM DTT, 5 mM ATP, 6.3 nM recombinant hGatCA (WT, G117E, or G133V), 1.03 μM recombinant hGatB, 65 nM [^14^C]Glu-tRNA^Gln^ and 2 mM glutamine. Over time, aliquots of the reaction mixture were taken at 0, 1, 5, 10, and 15 min, and were mixed with phenol–chloroform to extract aminoacyl-tRNAs under acidic conditions, followed by ethanol precipitation and removal of ATP using a Nap5 column. The amino acids attached to the tRNA were deacylated at 37°C for 30 min in 0.3% aqueous ammonia. The [^14^C] labeled amino acids were analyzed by thin-layer chromatography (TLC) on a cellulose plate (Melck) using a basic solvent system (28% ammonia solution:chloroform:methanol, 1:3:4). The TLC plate was exposed to an imaging plate, and the radioactivity was visualized using FLA-7000 image analyzer (Fujifilm).

### High-density oligonucleotide array methodology

Samples were processed in accordance with the manufacturer’s instructions. In brief, two aliquots of 250 ng genomic DNA were digested with Nsp1 and Sty1, and ligated to adaptors. Generic primers recognizing the enzyme-specific adaptor sequences were used to amplify adaptor-ligated DNA. After purification, 270 μg of the PCR product was fragmented and labeled with biotin. Hybridization was performed in an Affymetrix GeneChip Hybridization Oven 640, and the arrays were washed and stained in an Affymetrix GeneChip Fluidics Station 450. Arrays were scanned with an Affymetrix GeneChip Scanner 3000 7G. Hardware scripts were enabled and image processing performed using the Affymetrix GeneChip Command Console software (AGCC). Genotypes were called using the Affymetrix Genotyping Console software v4.1.1 GTC with the Birdseed algorithm and a default-calling threshold of 0.1. Samples were required to have an average minimum Quality Control SNP call rate of 99.7%.

### Copy number analysis

All samples were analyzed with GTC v4.1.1. The predicted copy numbers as well as the start and end of each CNV segment were determined using the Hidden Markov Model. In all datasets, hg19 was used. All large CNVs were manually curated. The CNV calls were also generated using the PennCNV software[68].

### Statistics

Results are presented as mean ± SEM or SD for the number of experiments indicated in the figure legends. Statistical analysis of continuous data was performed with 2-tailed Student’s t test, as appropriate. p < 0.05 was considered statistically significant.

### Study approval

The study was approved by the ethics committee of the Saitama Medical University. Written informed consent was obtained from all subjects prior to inclusion in this study.

### URLs

1000 Genomes Project, http://www.1000genomes.org/; hs37d5.fa ftp://ftp.1000genomes.ebi.ac.uk/vol1/ftp/technical/reference/phase2_reference_assembly_sequence/; DECIPHER, https://decipher.sanger.ac.uk; DGV, http://dgv.tcag.ca/dgv/app/home; ExAC [Dec., 2014 accessed], Cambridge, MA, http://exac.broadinstitute.org; ESP6500 [accessed via ANNOVAR 2013Feb21 version], http://evs.gs.washington.edu/EVS/; FASTQC, http://www.bioinformatics.babraham.ac.uk/projects/fastqc/; GERP, http://mendel.stanford.edu/SidowLab/downloads/gerp/; The Human Genetic Variation Database (HGVD), http://www.genome.med.kyoto-u.ac.jp/SnpDB/index.html; Hgvd2annovar, https://github.com/misshie/hgvd2annovar; Homologene, http://www.ncbi.nlm.nih.gov/homologene; MitoCarta http://www.broadinstitute.org/pubs/MitoCarta/index.html; mtDB, http://www.mtdb.igp.uu.se/; NextCODE, http://www.nextcode.com/; OMIM, http://www.omim.org; Picard, http://broadinstitute.github.io/picard/; PolyPhen-2, http://genetics.bwh.harvard.edu/pph2/; R for statistical analysis, http://www.R-project.org/; Ruby, https://www.ruby-lang.org/en/; SIFT, http://sift.jcvi.org/.

## Supporting Information

S1 Text Detailed description of identified mutations and variants in this study. (DOCX)

S1 Fig Characteristics of 142 unrelated patients with childhood-onset mitochondrial respiratory chain complex deficiencies. Histogram of enzymatic diagnoses in our cohort (**A**). Severity was categorized as “Major criteria” or “Minor criteria” on the basis of the level of enzyme activity reduction (**B**). Distribution of age at diagnosis (**C**). Histogram of clinical diagnoses in our cohort (**D**). MTDPS, mitochondrial DNA depletion syndrome; ETC, electron transport chain.
(TIF)

S2 Fig Flowchart of filtering variants detected by whole exome sequencing. (TIF)

S3 Fig Enrichment of prioritized genes in cases in comparison with those in healthy individuals. One hundred twenty-eight out of 142 cases, and 175 ethnically matched healthy controls whose sequence reads exceeded 50 million were included in the analysis. Percentages of cases and controls containing prioritized known OXPHOS disease-causing genes were elucidated using simplified exome analysis (**A**). Percentages of cases and controls containing prioritized mitochondria-related genes were elucidated using simplified exome analysis (**A**). Percentage of cases and controls containing 908 randomly selected other genes were elucidated using simplified exome analysis (**B**).
(TIF)

S4 Fig Large mtDNA deletion in Pt334. Schematic diagram of mtDNA indicates the deletion m.11359_15068del3710 (black arc) (**A**). Gel electrophoresis (1% agarose gel) of a long-range PCR amplicon shows an 8,701 bp fragment in control DNA and 4,992-bp fragment in Pt334; All Purpose Lo DNA Marker (BNX) and mtDNA with a deletion from other patient (Single del) are also included (**B**). mtDNA sequence coverage (**C**). Inset shows Sanger electropherogram of the breakpoint.
(TIF)

S5 Fig *BOLA3* mutations in Pt045, Pt268, Pt286, and Pt314. Family pedigrees and Sanger sequencing results for Pt045 (**A** and **B**), Pt268 (**C** and **D**), Pt286 (**E** and **F**), and Pt314 (**G** and **H**). Compound heterozygous mutations c.287A>G (p.H96R) and c.225_229del (p.K75fs) in *BOLA3* (NM_212552) were identified in Pt045. Pt268, Pt286, and Pt314 had a homozygous mutation c.287A>G (p.H96R) in *BOLA3* (NM_212552). Eu means uninformative DNA test. ClustalW alignment of BOLA3 orthologs. The residue p.H96 is highly conserved (**I**).
(TIF)

S6 Fig Complementation assay of Pt045, Pt268, Pt286, and Pt314 fibroblasts. The mitochondria were isolated from control or patient fibroblasts following the lentiviral-mediated expression of *mito-TurboRFP-V5* or *BOLA3-V5* cDNA and were analyzed by BN-PAGE/Western blotting and mitochondrial respiratory chain complex enzyme assays. Complementation with *BOLA3-V5* restored the assembly levels of both complexes (**A**,**D**,**G**,**J**) and enzyme activities (**C**,**F**,**I**,**L**) in all patient fibroblasts. mito-TurboRFP-V5 and BOLA-V5 proteins in the isolated mitochondria were detected by SDS-PAGE/Western blotting (**B**,**E**,**H**,**K**). HSP60 was used as a loading control. RFP, *mito-TurboRFP-V5*; CS, citrate synthase.
(TIF)

S7 Fig *NDUFAF6* mutations in Pt101, Pt330, Pt512, and Pt598. Family pedigrees and electropherograms for Pt101 (**A** and **B**), Pt330 (**C** and **D**), Pt512 (**E** and **F**), and Pt598 (**G** and **H**). The mutation c.371T>C in *NDUFAF6* (NM_152416) was shared between Pt101 and Pt598. The mutation c.805C>G was found in both Pt101 and Pt512. Eu means uninformative DNA test.
(TIF)

S8 Fig Gene structure of *NDUFAF6*, with gene product protein domains and identified mutation positions. (TIF)

S9 Fig Complementation assay in Pt330 and Pt512 fibroblasts. The mitochondria were isolated from control fibroblasts or Pt330 and Pt512 fibroblasts following the lentiviral-mediated expression of *mito-TurboRFP-V5*, *NDUFAF6*, or *NDUFAF6-V5* cDNA and were analyzed by BN-PAGE and Western blotting. Complementation with *NDUFAF6* or *NDUFAF6-V5* restored the assembly levels of complex I in patient fibroblasts (**A** and **C**). mito-TurboRFP-V5, NDUFAF6 and NDUFAF6-V5 proteins in the isolated mitochondria were detected by SDS-PAGE/Western blotting with anti-V5 and anti-NDUFAF6 antibodies (**B** and **D**). HSP60 was used as a loading control. RFP, *mito-TurboRFP-V5*. BN-PAGE of mitochondrial fractions from Pt101 and Pt598 fibroblast cells (**E** and **F**).
(TIF)

S10 Fig *NDUFB11* mutations in Pt067. Family pedigrees of Pt067. The hemizygous c.391G>A mutation was *de novo* (**A**). Electropherograms for Pt067 (**B**). A hemizygous mutation c.361G>A (p.E121K) in *NDUFB11* (NM_001135998) was found in Pt067. Eu means uninformative DNA test. ClustalW alignment of NDUFB11 orthologs shows conservation of the p.E121 residue (**C**). SDS-PAGE/Western blotting analysis showed a decrease in the endogenous NDUFB11 protein level in Pt067 fibroblasts. HSP60 was used as a loading control (**D**). siRNA knockdown of NDUFB11 in normal fibroblasts. BN-PAGE/Western blotting of mitochondrial fractions derived from normal human fibroblasts transfected with si*NDUFB11* (**E**). *NDUFB11* mRNA and NDUFB11 protein expression in cells treated with siRNAs targeting *NDUFB11* (Clone #94, #95 and #96) and control cells (siNega #2) were confirmed by quantitative real-time PCR and SDS-PAGE/Western blotting, respectively (**F** and **G**).
(TIF)

S11 Fig RNAi-mediated *dndufb11* (*NP15*.*6*) knockdown in *Drosophila melanogaster*. Schematic representation of *dndufb11* (*NP15*.*6*) and *dgatA* in *Drosophila melanogaster* (**A**). *dndufb11* is located in intron 3 of *dgatA*, a *QRSL1* ortholog. Quantitative PCR revealed dramatically reduced *dndufb11* mRNA levels in RNAi-generated *dndufb11*-knockdown flies (*arm > dndufb11-IR*) to approximately 24% of control levels (*arm > GFP-IR*), whereas *dgatA* mRNA levels were normal (**B**). All relative values were calculated against data from control flies. Climbing activities of *arm > dndufb11-IR* flies and control flies at the age of 6 days (**C**). Significance relative to controls was calculated using Student’s t test (*; p < 0.01; n.s., not significant). The longevity of *arm>dndufb11-IR* male flies was dramatically reduced relative to that of control flies (log-rank test; p < 0.01) (**D**). Metabolic rate was estimated from CO_2_ production (μl CO_2_/mg weight/h) of 4-day-old male flies (**E**). BN-PAGE of mitochondrial fractions from *arm>dndufb11-IR* and control male flies (**F**). The mitochondria were resuspended in native-PAGE sample buffer with 1% digitonin. Wedges indicate increasing amounts of mitochondrial extract (10 μg, 25 μg, 50 μg). Relative amounts of lactate and pyruvate in *dndufb11-*knockdown flies against control flies (**G**). The amounts of metabolites were determined by LC-MS analysis. Data represent mean ± SEM of at least three experiments. Student's t-test was used to compare the data between *dndufb11-*knockdown and control (*arm>GFP-IR*) flies. (*; p < 0.01).
(TIF)

S12 Fig *KARS* mutations in Pt459. Family pedigrees of Pt459 (**A**). Electropherograms for Pt459 (**B**). Compound heterozygous mutations c.1343T>A (p.V448D) and c.953T>C (p.I318T) in *KARS* (NM_005548) found in Pt459. ClustalW alignment of KARS orthologs shows conservation of both the p.V448 and p.I318 residues (**C**). mito-TurboRFP-V5, mtKARS-V5 (mitochondrial isoform of KARS), and cytKARS-V5 (cytosolic isoform of KARS) proteins in isolated mitochondria were detected by SDS-PAGE/Western blotting with an anti-V5 antibody (**E**). HSP60 was used as a loading control. Respiratory chain complex enzyme activity was measured twice; activity is shown as a percentage of citrate synthase activity. Enzyme activities (**D**) and complex I and IV assembly (**F**) were rescued in Pt459 fibroblasts by the overexpression of wild-type mitochondrial *KARS* cDNA. RFP, *mito-TurboRFP-V5*; mt-KARS, mitochondrial *KARS* (NM_001130089)*-V5*; cyt-KARS, cytosolic *KARS* (NM_005548)*-V5*; CS, citrate synthase.
(TIF)

S13 Fig *MRPS23* mutations in Pt276. Electropherograms for Pt276 (**A**). A homozygous mutation c.119C>G (p.P40R) in *MRPS23* (NM_016070) was found in Pt276. ClustalW alignment of *MRPS23* orthologs shows conservation of the p.P40 residue (**B**). MRPS23-V5 protein expression in *MRPS23*-V5-overexpressing Pt276 cell lines was confirmed by SDS-PAGE/Western blotting with an anti-V5 antibody (**C**). RFP, *mito-TurboRFP-V5*.
(TIF)

S14 Fig *QRSL1* mutations in Pt250 and Pt860. Electropherograms for Pt250 (**A**) and Pt860 (**B**). Homozygous and compound heterozygous mutations c.398G>T (p.G133V) and c.350G>A (p.G117E) in *QRSL1* (NM_018292) found in Pt250 and Pt860, respectively. Sequence alignment of QRSL1 orthologs (**C**).
(TIF)

S15 Fig *MECP2* mutations in Pt053 and Pt369. Family pedigree and electropherograms for Pt053 (**A** and **B**). The c.806delG (p.G269fs) mutation in *MECP2* (NM_004992) has been reported in males with severe neonatal encephalopathy. Family pedigree and electropherograms for Pt369 (**C** and **D**). Another novel mutation in *MECP2* (NM_001110792) c.17_18insG (p.A6fs) was identified in Pt369. SDS-PAGE/Western blotting analysis exhibited loss of the endogenous MECP2 protein level in isolate nuclear fractions from Pt053 and Pt369 fibroblasts and MECP2 knockdown fibroblasts (**E**). Lamin A/C was used as a loading control.
(TIF)

S16 Fig *TNNI3* mutation in Pt827. Family pedigree for Pt827 (**A** and **B**). A heterozygous mutation c.575G>A (p.R192H) in *TNNI3* (NM_000363), which was previously identified in patients with restrictive cardiomyopathy, was confirmed *de novo* via Sanger sequencing of the parents’ DNA. The transmission electron microscope image of the mitochondria in a cardiac muscle tissue of normal individual and the patient (**C**). The mitochondria were often enlarged with increased cristae density. Scale bar, 500 nm.
(TIF)

S17 Fig *De novo* chromosomal microdeletions identified in patients with mitochondrial respiratory chain deficiency. *De novo* deletions of 6q24.3-q25.1 were identified using a high-density oligonucleotide array in Pt452 and Pt695 (**A**). *De novo* deletion of 22q11.21 was identified using a high-density oligonucleotide array in Pt587 (**B**).
(TIF)

S1 Table Detailed clinical information. CI, complex I deficiency; CIII, Complex III deficiency; CIV, complex IV deficiency; CC, combined complex deficiencies; CM, cardiomyopathy; EP, enteropathy; HD, hepatic disease; LD, Leigh's disease; LIMD, lethal infantile mitochondrial disorder; MC, mitochondrial cytopathy; ND, neurodegenerative disorder; NLIMD, non-lethal infantile mitochondrial disorder; SS, short stature; SUD, sudden unexpected death; CHD, congenital heart defect(s); COX, cytochrome c oxidase; CPA, cardiopulmonary arrest; DD, developmental delay; FTT, failure to thrive; HCM, hypertrophic cardiomyopathy; IUGR, intrauterine growth restriction; MOF, multiple organ failure; MRI: magnetic resonance imaging; RRF, ragged red fiber; SSV, strongly SDH-reactive blood vessel.
(XLSX)

S2 Table Statistics of sequence reads. CCDS, consensus cDNA (downloaded on 2012.11.14).
(XLSX)

S3 Table Mutations and pVUS in nuclear DNA. Detailed list of all prioritized variants and all confirmed pathogenic mutations identified from WES in this study de_novo, *de novo* mutation confirmed by trio-based Sanger sequencing; sib_segregate, The variant is segregated with phenotypes between sibling(s); trio_confirmed_by_Sanger, the variant is confirmed by trio-based Sanger sequencing; validated_by_cDNA_phasing, phased using cDNA; validated_by_gDNA_phasing, phased using gDNA; validated_by_sanger, the variant is validated by Sanger sequencing; chrX_male_mother, the variant is inherited from mother to son not_clear_signal_by_sanger, the variant is not validated well by Sanger sequencing.
(XLSX)

S4 Table Mutations and pVUS in mitochondrial DNA. rCRS, revised Cambridge Reference Sequence (NC_012920); CI, complex I deficiency; CIV, complex IV deficiency; CC, combined complex deficiencies; LD, Leigh's disease; LIMD, lethal infantile mitochondrial disorder; MC, mitochondrial cytopathy; NLIMD, non-lethal infantile mitochondrial disorder; SUD, sudden unexpected death; LR-PCR, long-range PCR; mtDB, Human Mitochondrial Genome Database.
(XLSX)

S5 Table Pathogenic CNV and pVUS in our cohort. All positions are based on hg19 reference sequences. *De novo*, confirmed *de novo* mutation by trio-based copy number analysis; N.A., not available.
(XLSX)

S6 Table List of quantitative RT-PCR primers. (XLSX)

S7 Table List of primers used for sequence validation. (XLSX)

S8 Table List of cDNA cloning primers. (XLSX)
