# Supplementary material for: A Comprehensive Genomic Analysis Reveals the Genetic Landscape of Mitochondrial Respiratory Chain Complex Deficiencies
Source: PLoS Genet. 2016 Jan 7;12(1):e1005679. doi: 10.1371/journal.pgen.1005679 (PMC4704781; doi:10.1371/journal.pgen.1005679)
Supplement: S1 Text — (DOCX) [file pgen.1005679.s001.docx]

S1 Text. Detailed description of identified mutations and variants in this study

**Mutations in mitochondrial DNA.** We searched for variants annotated as confirmed or reported in MITOMAP and large deletions detected through mitochondrial genome mapping and *de novo* mtDNA assembly. We identified confirmed mutations in 9 (6.3%) patients (S4 Table). We also discovered a 3.7-kb deletion in the mitochondrial genome of Pt334, in whom no other strong candidate genes were identified via whole-exon analysis. This deletion (m.11359_15068del3710) was not reported previously and was estimated to be present in >95% of the total mitochondrial DNA (S4 Fig.).

**pVUS in mitochondrial DNA.** Variants categorized as reported in MITOMAP and suspected of pathogenicity are listed in S4 Table as pVUS. We curated the pathogenicity of these variants and found that the m.3946G>A variant was very likely to be pathogenic because it was found in 2 independent patients (Pt619, Pt640) and was proven to be pathogenic using a cybrid assay[69]. The 0% allele frequency in mtDB[70] and inability to find prioritized variants during exome analysis further support this mtDNA variant as a causative variant.

**Support for pathogenicity of known mitochondrial disease-causative genes**

***ACAD9* mutations.** *ACAD9*, which encodes acyl-CoA dehydrogenase-9 and is the complex I assembly factor, was mutated in 2 unrelated patients and their sisters. Pt025 and Pt090 exhibited hypertrophic cardiomyopathy and complex I deficiency (MIM 611126). Pt025 and her elder sister with mitochondrial respiratory chain deficiency harbored the compound heterozygous mutations c.811T>G (p.C271G) and c.1766-2A>G inherited from their father and mother, respectively. Her healthy younger brother was shown to have wild-type variants in *ACAD9*. Pt090 harbored the compound heterozygous mutations c.1150G>A (p.V384M) and c.1817T>A (p.L606H). Both the p.V384, and p.L606 residues are highly conserved and these two mutations were predicted to be “probably damaging” by the PolyPhen2 and SIFT algorithms. Western blotting revealed a severe decrease in ACAD9 protein levels in fibroblast cells from Pt025. *ACAD9* overexpression rescued the biochemical complex I abnormalities in Pt025 fibroblast cells.

***COQ4* mutations.** Pt113, a girl with intrauterine growth restriction, hypertrophic cardiomyopathy, respiratory failure, and combined respiratory chain deficiencies (I and III), harbored compound heterozygous mutations c.718C>T (p.R240C) and c.421C>T (p.R141X) in *COQ4*. Her father and mother were heterozygous carriers of c.421C>T and c.718C>T, respectively. The p.R240 residue is highly conserved and mutation is predicted to be “probably damaging” by the PolyPhen2 and SIFT algorithms. Although Pt113 is the first case harboring mutations in *COQ4*, she is included in our recent paper by Brea-Calvo et al[11]. In our previous report of COQ4, the p.141X, p.R240C, and a combination thereof failed to rescue a COQ4-null strain in a yeast model, indicating that these mutations lead to a loss of COQ4 function.

***COX10* mutations.** COX10, which encodes a cytochrome c oxidase assembly protein, was mutated in Pt223, who presented with hypertrophic cardiomyopathy, renal failure, and combined respiratory chain deficiencies (I, II, and IV). Pt223 harbored the compound heterozygous nonsynonymous mutations c.1259C>T (p.P420L) and c.862G>A (p.G288R) . A ClustalW alignment of COX10 orthologs showed that the p.G288 and p.P420 residues are highly conserved and these two mutations were predicted to be “probably damaging” by PolyPhen2 and SIFT. We performed a complementation experiment to assess whether the introduction of wild-type *COX10* cDNA into the patient’s fibroblasts would rescue these defects in complex IV activities. The expression of wild-type *COX10* rescued the complex activities and protein levels in fibroblasts from Pt223, thereby establishing *COX10* as the causal gene in this case.

***EARS2* mutations.** The compound heterozygous mutations c.319C>T (p.R107C) and c.1466G>A (p.R489Q) in *EARS2* were observed in Pt691, a boy with seizures, myopathy, developmental delays, and complex IV deficiency. His mother was shown to be heterozygous for the c.1466G>A, whereas his father only carried wild-type *EARS2* alleles. One mutation, c.319C>T, was confirmed to be *de novo*. c.319C>T (p.R107C) is likely to be associated with the disease because c.320G>A (p.R107H) is reported as a known disease-causing allele[71]. These mutations were phased by cDNA sequencing. A ClustalW alignment of EARS2 orthologs shows conservation of both the p.R107 and p.R489 residues and these two mutations were predicted to be “probably damaging” by the PolyPhen2 and SIFT algorithms.

***ECHS1* mutations.** *ECHS1* (NM_004092) is a recently reported causative gene for Leigh’s disease[72,73]. We independently identified two patients (Pt346 and Pt376) harboring a c.176A>G (p.N59S) mutation in this gene. Another mutation, c.476A>G (p.Q159R), was observed in Pt346 and is reportedly shared by two independent foreign families with similar clinical phenotypes[12]. They were included in our recent papers by Haack et al[12]. In Pt346, we also identified a de novo mutation, c.471G>C (p.L157F), in POLG2 (NM_007215), but this de novo mutation is not shared with a affected sibling. We concluded that POLG2 is not the primary causative gene for this family.

***GFM1* mutations.** *GFM1*, which encodes the mitochondrial elongation factor G1, was mutated in a boy (Pt112) with microcephalus, seizures, liver failure, developmental delay, and combined respiratory chain deficiencies (I, III, and IV). Pt112 harbored compound heterozygous mutations c.170C>A (p.S57Y) and c.748C>T (p.R250W). The variant c.748C>T is a known[13] mutation.

***GTPBP3* mutations.** *GTPBP3*, which encodes a GTPase involved in taurinomethyluridine modification of the wobble uridine base in mitochondrial tRNAs, was mutated in Pt751, a girl with severe hypotonia, developmental delays, lactic acidosis, and combined respiratory chain deficiencies (I and IV). She harbored the compound heterozygous mutation c.8G>T (p.R3L and 25-bp deletion c.923-947del (p.E309fs). Although Pt751 is the first case harboring mutations in *GTPBP3*, she is included in our recent papers by Kopajtich et al[14].

***MPV17* mutations.** *MPV17*, which encodes a mitochondrial inner membrane protein and is one of the known causal genes for mitochondrial depletion syndrome, was mutated in a girl (Pt339) with acute encephalopathy, liver dysfunction, failure to thrive, hypoglycemia, and combined respiratory chain deficiencies (I and III). Pt339 harbored compound heterozygous mutations c.293C>T (p.P98L) and c.376-1G>A. c.293C>T was a reported mutation in hepatocerebral mitochondrial DNA depletion syndrome[15]. The mtDNA decreased to 20.5% in hepatic tissue, which is consistent with the phenotype of mitochondrial DNA depletion syndrome 6 (hepatocerebral type) (MIM 256810) caused by mutated *MPV17*.

***NDUFA10* mutations.** *NDUFA10* mutations were reported in a boy with Leigh’s disease because of mitochondrial complex I deficiency[74]. Pt057 presented with intrauterine growth retardation, lactic acidosis, pulmonary hypertension, and complex I deficiency. This patient harbored the compound heterozygous mutations c.881T>C (p.L294P) and c.383_384insTAA (p.S128delinsIS), inherited from his father and mother, respectively. A ClustalW alignment of NDUFA10 orthologs indicated conservation of the p.L294 residue and, the SIFT algorithm predicted the p.L294P mutation to be “damaging”. SDS-PAGE/Western blotting analysis indicated a decrease in the endogenous NDUFA10 protein level in mitochondria isolated from Pt057 fibroblasts. Complementation with wild-type *NDUFA10* restored the complex I assembly.

***NDUFS4* mutations.** Pt711, a girl with myocarditis, respiratory failure, delirium, basal ganglia abnormalities on brain MRI, and complex I deficiency, harbored the homozygous mutations c.340T>C (p.W114R) in *NDUFS4*. Her parents were heterozygous carriers of c.340T>C. We confirmed a high level of p.W114 residue conservation, and the PolyPhen2 and SIFT algorithms predicted the observed p.W114R change to be “probably damaging”. A high-density oligonucleotide array analysis identified an approximately 6.2Mb LCSH encompassing *NDUFS4*. No other candidate gene was prioritized in our comprehensive genomic analysis. Mutations in *NDUFS4* have been reported to induce the accumulation of high levels of lower molecular weight subcomplexes[75] in BN-PAGE profiles. However, we did not observe similar subcomplexes in fibroblasts from Pt711, despite an apparent decrease in the amount of complex I. This difference may have been caused by the use of digitonin to solubilize mitochondria in the previous study; Triton X-100 was used in the present study.

***RARS2* mutations.** Mutations in *RARS2,* which encodes mitochondrial arginyl-transfer RNA synthetase, have been reported in several patients with pontocerebellar hypoplasia type 6 (MIM 611523). Pontocerebellar hypoplasia is a heterogeneous group of severe developmental disorders characterized by early-onset seizures, progressive microcephaly, and developmental delays. Pt222 displayed lactic acidosis, cerebral atrophy, seizures, developmental delays, and complex IV deficiency. The compound heterozygous mutations c.1306G>T (p.D436Y) and c.1321C>T (p.L441F) were identified in this patient and his symptomatic younger brother. The c.1306G>T and c.1321C>T mutations were inherited from her father and mother, respectively. Both the p.L441 and p.D436 residues were highly conserved. The p.D436Y mutation is predicted to be “probably damaging” by PolyPhen2 and SIFT, whereas the p.L441F mutation is predicted to be “probably damaging” only by the PolyPhen2 algorithm. This patient presented with progressive brain cortical atrophy, severe epilepsy, marked developmental delays, lethargy, and pontocerebellar hypoplasia with neonatal onset, all of which were similar to the clinical features of patients reported to harbor mutations in *RARS2*.

***RRM2B* mutations.** The homozygous mutation c.97C>T (p.P33S) in *RRM2B* was identified in Pt652, who presented with muscle weakness, hearing loss, and combined respiratory chain deficiencies (I and IV). High-density oligonucleotide array analysis identified an approximately 1.1Mb LCSH encompassing *RRM2B*. There was no evidence of a deletion a CNV or an exonic confirmed using array and WES data. This homozygous mutation was shared with her symptomatic younger sister; whereas her healthy oldest and youngest sisters were heterozygous. Her mother was a heterozygous carrier of c.97C>T; a DNA sample from the father was not available. Although the same homozygous variant was previously reported[76] and recorded as a pVUS in OMIM (http://omim.org/entry/604712#0014) in a Japanese patient with a similar phenotype, we consider this to be a pathogenic mutation causative of mitochondrial respiratory chain deficiency.

***SCO2* mutations.** SCO2 is essential for assembly of the catalytic core of cytochrome-c oxidase (COX) in complex IV. Mutations in *SCO2* have been associated with fatal infantile cardioencephalomyopathy because of cytochrome c oxidase deficiency (MIM 604377). We identified the compound heterozygous mutations c.773T>C (p.M258T) and c.577G>A (p.G193S) in dichorionic diamniotic twins (II:1[Pt628] and II:2). Mutation c.577G>A (p.G193S) was previously reported to be pathogenic[16]. A ClustalW alignment of the SCO2 orthologs indicated conservation of the p.M258 residue, and the PolyPhen2 and SIFT algorithms predicted the p.M258T change as “probably damaging”. The father of this patient was shown to be a heterozygous carriers of c.577G>A, and his mother was heterozygous for c.773T>C. The twins presented with intrauterine growth retardation, premature birth, cranial abnormality (via magnetic resonance imaging), and lactic acidosis. Complex I and IV deficiencies were detected in twin II:1 (Pt628), but not II:2.

***SUCLA2* mutations.** *SUCLA2*, which encodes mitochondrial succinyl-CoA ligase subunit beta, was mutated in a boy with developmental delay, myopathy, ptosis, and complex IV deficiency. Pt105 harbored a homozygous known mutation[17] c.1048G>A (p.G350S), located in 44.4Mb LCSH. High-density oligonucleotide arrays confirmed no evidence of a large deletion surrounding this LCSH.

***TAZ* mutation.** *TAZ* encoding tafazzin is involved in the metabolism of cardiolipin that is implicated in the formation of mitochondrial respiratory chain complex. Barth syndrome with cardiomyopathy, neutropenia, growth retardation, and 3-methylglutaconic aciduria caused by a mutation in the *TAZ* gene (MIM 302060). Pt634 displayed asphyxia, hypotonia, developmental delay, failure to thrive, and combined respiratory chain deficiencies (I and IV). Pt634 harbored a hemizygous mutation c.36_57del (p.V12fs) in *TAZ* and the deafness-associated m.1555A>G homoplasmic variant in RNR1. The m.1555A>G variant was unlikely to be pathogenic, given that Pt634 did not show deafness. We found a loss of endogenous TAZ protein level in total cell lysates of Pt634 fibroblasts. We accordingly hypothesize that *TAZ* is causative of mitochondrial respiratory chain deficiency.

***TUFM* mutations.** The gene encoding TUFM, which is required for translational elongation in mitochondria, was mutated in 2 unrelated patients with intrauterine growth restriction, respiratory failure, hypotonia, and either complex IV deficiency (Pt559) or combined respiratory chain deficiencies (I, III, and IV; Pt622). Pt559 carried the homozygous mutation c.440T>A (p.L147H) within an 8.0Mb LCSH. Sanger sequencing identified the parents to be the heterozygous carriers of this mutation. This c.440T>A mutation was shared with Pt622, who also harbored the previously reported pathogenic mutation c.162delC (p.Y54X)[77]. A symptomatic younger brother of Pt559 harbored the same *TUFM* mutations as the proband. A symptomatic younger sister of Pt622 harbored the same compound heterozygous *TUFM* mutations as the proband. The compound heterozygous mutations c.440T>A and c.162delC were inherited from their father and mother, respectively. The residue p.L147 is highly conserved and p.L147H was predicted to be “damaging” by the SIFT algorithm. Complementation with wild-type *TUFM* restored the complex I and IV assembly and complex IV activity levels in fibroblasts from Pt622.

**pVUS in known OXPHOS disease-causing genes**

***PC* pVUS.** Pt001, a girl with congenital hydrocephalus, lactic acidosis, and combined respiratory chain deficiencies (I and IV), had compound heterozygous variants c.1822G>A (p.G608R) and c.2120C>T (p.T707M) in the *PC* gene (NM_022172). The p.G608 and p.T707 residues are highly conserved among species, and related two mutations are predicted to be “probably damaging” by the PolyPhen2 and SIFT algorithms. Compound heterozygous variants in *PC* were confirmed by cDNA sequencing to be localized in a separate allele (phased). Pyruvate carboxylase is a nuclear-encoded mitochondrial enzyme that catalyzes the conversion of pyruvate to oxaloacetate. We considered a diagnosis of pyruvate carboxylase deficiency for this patient. Taylor et al[40] also identified *PC* variants associated with multiple mitochondrial respiratory chain defects (CI and CIV). We consider that the complex I and IV deficiencies were caused by secondary, because of the lack of direct molecular evidence related to OXPHOS.

***LRPPRC* pVUS.** *Leucine-rich pentatricopeptide repeat motif-containing protein, mitochondrial (LRPPRC)* encodes an RNA-binding protein that stabilizes the polyadenylated mitochondrial mRNA[78]. Pt550, a girl with multiple organ failure, lactic acidosis, and combined respiratory chain deficiencies (I and IV), harbored compound heterozygous variants c.1253A>C (p.N418T) and c.2741C>A (p.P914Q). The p.N418 and p.P914 residues are highly conserved between species. The PolyPhen2 and SIFT algorithms is predicted p.P914Q to be “probably damaging” and p.N418T to be “benign” or “tolerated”. The compound heterozygous mutations c.1253A>C and c.2741C>A were inherited from her father and mother, respectively. Mutations in *LRPPRC* have been reported in French Canadian Leigh syndrome (MIM 220111). Although we do not have direct evidence to support the pathogenesis of compound heterozygous variants in Pt550 yet, we suspect *LRPPRC* to be causative of mitochondrial respiratory chain deficiency.

**pVUS in mitochondria-related genes**

***ALAS2* pVUS.** Pt125 harbored a hemizygous variant c.613C>T (p.R205C) in *ALAS2* (NM_001037968). Phenotypic aberrations in *ALAS2* have been listed in OMIM and referred to as anemia, sideroblastic, X-linked (MIM 300751) and protoporphyria, erythropoietic, X-linked (MIM 300752). We consider that this *ALAS2* variant will unlikely be pathogenic because this proband did not have a similar clinical presentation.

***C1QBP* pVUS.** Pt273, a girl with intrauterine growth restriction, heart failure, respiratory failure, and combined respiratory chain deficiencies (I, III, and IV), harbored the compound heterozygous variants pVUS c.739G>T (p.G247W) and c.824T>C (p.L275P) in *C1QBP*. C1QBP plays a role in mitochondrial translation and is involved in the formation of functional 55S mitoribosomes and nucleolar ribosome maturation. After subjecting the sequence data of this single proband a to detailed bioinformatic analysis under a recessive model, prioritization of all detected sequence variants led to the consideration of a single candidate as the causative genetic defect in this proband. *C1QBP*-deficient mice exhibit mid-gestation lethality associated with a severe embryonic developmental defect. Primary embryonic fibroblasts isolated from *C1QBP* knockout embryos also exhibit severe combined respiratory chain deficiencies (I, III, and IV) because of severely impaired mitochondrial protein synthesis[79]. Although we do not have direct evidence to support the pathogenesis of the compound heterozygous variants in Pt273, we suspect *C1QBP* to be causative of mitochondrial respiratory chain deficiency.

***FAM65C* pVUS.** Pt827, who harbored a *de novo* mutation in *TNNI3*, also harbored compound heterozygous variants c.1139T>G (p.L380R) and c.1954G>A (p.E652K) in *FAM65C* (NM_080829), which is included in the MitoCarta database. We consider *TNNI3* to be the primary cause in this patient on the basis of the phenotypic similarity and *de novo* status of the mutation.

**pVUS in non-mitochondria-related genes**

***CLCN2* pVUS.** Pt546 was suspected of metachromatic leukodystrophy or Krabbe leukodystrophy with complex I deficiency. We identified compound heterozygous variants c.62_63insC (p.L21fs) and c.1828C>T (p.R610X) in *CLCN2* (NM_004366) that have been reported as a causative gene for leukodystrophy[80,81]. Taken together, patient was diagnosed with leukodystrophy with ataxia (OMIM: 615651). His father and mother were a heterozygous carries of c.1828C>T and c.62_63insC. We consider the complex I deficiency have been caused secondarily, given the lack of direct molecular evidence related to mitochondrial function.

***TTC37* pVUS.** Similarly to his deceased sister, Pt529, a boy with complex I deficiency, exhibited kinky hair, a fair complexion, chronic diarrhea, and failure to thrive. The reduction of ETC activity in this patient was classified as major. He was found to harbor c.196_197insA (p.A66fs) and c.1135-1G>T variants in *TTC37* by exome analysis. c.1135-1G>T was located in intron 13 and caused a loss of the original splice site, leading to the use of a novel acceptor site. *TTC37* mRNA and protein expression was apparently decreased. *TTC37* causes trichohepatoenteric syndrome 1 (MIM 222470), which is characterized by a phenotype similar to that of our patient. Taken together, this patient was diagnosed with trichohepatoenteric syndrome 1. Although we consider the complex I deficiency was caused by secondary, the exact relationship between tricohepatoenteric syndrome and mitochondrial disorders is yet to be elucidated.

**Copy number variations (CNV)**

***STS* CNV.** Pt133, a boy with ichthyosis and complex I deficiency, harbored an Xp22.31 deletion. This region contains *STS*, which is known to correlate with ichthyosis. Although we assume that this mutation explains the patient’s clinical phenotype, we cannot conclude that it is the sole mutation contributing to mitochondrial enzyme deficiency.

***GYG2* CNV.** In this study, we identified 2 male cases Pt057 and Pt587 with complete *GYG2* loss following a 102-kb deletion in Xp22.33 using high-density oligonucleotide array analysis. Although a hemizygous variant of *GYG2* was reported to be suggestive of the causative gene in Leigh’s disease[82], we do not conclude that this gene is causative in our cases because other confirmed causative mutations (*NDUFA10* and 22q11.21) were identified in both cases.

**Mutations in dominant-acting genes.** After removing variants using functional annotation filterings, we detected 8 candidate heterozygous variant alleles in 6 genes that may cause dominant-acting OXPHOS disease: *POLG* (Pt019 and Pt756), *RRM2B* (Pt192 and Pt598), *SLC25A4* (Pt298), *POLG2* (Pt346), *OPA1* (Pt363), and *C10orf2* (Pt639). We identified that Pt346 harbored the *de novo* mutation c.471G>C (p.L157F) in *POLG2* (NM_007215), but not shared with an affected sibling. Two alleles in Pt639 and Pt756 were inherited from the parents. DNA samples from the parents of Pt019, Pt192, Pt298, and Pt363 were not available. In Pt019, the c.2992T>G (p.S998A) mutation in *POLG* (NM_001126131) exhibited a higher allele frequency of 0.0679% (75 individuals recorded as heterozygotes) in the ExAC database. We consider this variant to be harmless. The remaining three variants were c.485C>G (p.S162C) in *RRM2B* (NM_001172477), Pt192; c.238C>A (p.R80S), in *SLC25A4* (NM_001151), Pt298; and c.1556T>C (p.M519T) in *OPA1* (NM_130831), Pt363. We could not determine the pathogenicity of these variants because these patients did not have sufficient molecular evidence or present with typical phenotypes. The *RRM2B* variant in Pt598 was not validated because the patient harbored mutations in *NDUFAF6*.

**Analysis of variants reported previously as pathogenic/suspected**

***FBN1* variant.** We also identified the known mutation c.3128A>G (p.K1043R) of *FBN1* (NM_000138)[83] in Pt185. Although this mutation (rs137854471) has been reported to cause neonatal Marfan syndrome (MIM 154700), the original report did not sequence trios (patient and parents) of DNA. Here we concluded this variant is benign because it was inherited from the healthy mother.

***FRMD7* variant.** We identified the known hemizygous mutation c.436C>T (p.R146W) in *FRMD7* (NM_194277) in Pt592. Although this mutation (rs137852211) has been reported to cause nystagmus 1, congenital, X-linked (MIM 300628), we concluded that this variant was not likely to cause mitochondrial respiratory chain deficiency because Pt592 did not have a phenotype similar to those reported previously.

***POR* variants.** We identified 2 known mutations, c.1237G>A (p.G413S) and c.601C>T (p.Q201X), of *POR* (NM_000941) in Pt498. Although these mutations, recorded in HGMD as CM057353 and CM068139, have been reported to cause disordered steroidogenesis due to cytochrome P450 oxidoreductase (MIM 613571), the original article[84] reported that p.G413S only had a small effect on the enzymatic activities of POR. Therefore, we concluded that these variants were unlikely to be causative in Pt498.

***MECP2* variant.** We identified the c.1133C>T (p.A378V) mutation of *MECP2* (NM_004992) in Pt593. Although 1133C>G (p.A378G) has been reported to cause Rett syndrome (MIM 312750), c.1133C>T was classified as a non-causative polymorphism in RettBASE (http://mecp2.chw.edu.au). Therefore, we concluded that this variant was unlikely to be the causative agent in Pt593.

**Validation of candidate genes that were filtered out in the current pipeline with stringent condition.** Our filtering strategy might have filtered out true pathogenic mutations. To validate the stringency of variant filtering, we performed relaxed allele frequency filtering (MAF in ESP> 1%, 1000 Genomes Project > 1% and HGVD > 1%). We evaluated if the true variants were included in those remained after relaxed filtering condition. We focused on 5 remaining genes (*ABCB7*, *HCCS,* *COX10*, *EARS2* and *ECHS1*) that cause mitochondrial disorder in recessive mode of trait. *ABCB7* gene in Pt101 was rejected, because the sister, presenting similar phenotype, had different genotype. *HCCS* in Pt286 was also denied because the patients harbored the confirmed mutation in *BOLA3*, and *HCCS* cDNA complementation assay failed to restore the enzymatic activities and respiratory chain complex formation. We identified support of evidence for *ECHS1* in Pt346, *COX10* in Pt223, and *EARS2* in Pt691, respectively (see Results and Support of pathogenicity in Supplemental information). *COX10* was dropped from the mainstream pipeline because one of the variants is located within a segmental duplication region. *EARS2* was dropped because one of the variants did not exceed the GERP score. *ECHS1* was dropped because one of the variants did not exceed the SIFT score. To avoid filtering out these variants, we also performed a mainstream pipeline without a segmental duplication filter and extracted variants in known mitochondrial disorder-causing genes and mitochondria-related genes.

**Supplemental References**

69. Kirby DM, McFarland R, Ohtake A, Dunning C, Ryan MT, Wilson C, et al. (2004) Mutations of the mitochondrial ND1 gene as a cause of MELAS. J Med Genet 41: 784–789. doi:10.1136/jmg.2004.020537

70. Ingman M, Gyllensten U (2006) mtDB: Human Mitochondrial Genome Database, a resource for population genetics and medical sciences. Nucleic Acids Res 34: D749–D751. doi:10.1093/nar/gkj010

71. Steenweg ME, Ghezzi D, Haack T, Abbink TEM, Martinelli D, van Berkel CGM, et al. (2012) Leukoencephalopathy with thalamus and brainstem involvement and high lactate “LTBL” caused by EARS2 mutations. Brain 135: 1387–1394. doi:10.1093/brain/aws070

72. Peters H, Buck N, Wanders R, Ruiter J, Waterham H, Koster J, et al. (2014) ECHS1 mutations in Leigh disease: a new inborn error of metabolism affecting valine metabolism. Brain 137: 2903–2908. doi:10.1093/brain/awu216

73. Sakai C, Yamaguchi S, Sasaki M, Miyamoto Y, Matsushima Y, Goto Y-I (2015) ECHS1 mutations cause combined respiratory chain deficiency resulting in Leigh syndrome. Hum Mutat 36: 232–239. doi:10.1002/humu.22730

74. Hoefs SJG, van Spronsen FJ, Lenssen EWH, Nijtmans LG, Rodenburg RJ, Smeitink JAM, et al. (2010) NDUFA10 mutations cause complex I deficiency in a patient with Leigh disease. Eur J Hum Genet 19: 270–274. doi:10.1038/ejhg.2010.204

75. Ugalde C, Janssen RJRJ, van den Heuvel LP, Smeitink JAM, Nijtmans LGJ (2004) Differences in assembly or stability of complex I and other mitochondrial OXPHOS complexes in inherited complex I deficiency. Hum Mol Genet 13: 659–667. doi:10.1093/hmg/ddh071

76. Takata A, Kato M, Nakamura M, Yoshikawa T, Kanba S, Sano A, et al. (2011) Exome sequencing identifies a novel missense variant in RRM2B associated with autosomal recessive progressive external ophthalmoplegia. Genome Biol. 12: R92. doi:10.1186/gb-2011-12-9-r92

77. Valente L, Tiranti V, Marsano RM, Malfatti E, Fernandez-Vizarra E, Donnini C, et al. (2007) Infantile encephalopathy and defective mitochondrial DNA translation in patients with mutations of mitochondrial elongation factors EFG1 and EFTu. Am J Hum Genet 80: 44–58. doi:10.1086/510559

78. Chujo T, Ohira T, Sakaguchi Y, Goshima N, Nomura N, Nagao A, et al. (2012) LRPPRC/SLIRP suppresses PNPase-mediated mRNA decay and promotes polyadenylation in human mitochondria. Nucleic Acids Res 40: 8033–8047. doi:10.1093/nar/gks506

79. Yagi M, Uchiumi T, Takazaki S, Okuno B, Nomura M, Yoshida S-I, et al. (2012) p32/gC1qR is indispensable for fetal development and mitochondrial translation: importance of its RNA-binding ability. Nucleic Acids Res 40: 9717–9737. doi:10.1093/nar/gks774

80. Depienne C, Bugiani M, Dupuits C, Galanaud D, Touitou V, Postma N, et al. (2013) Brain white matter oedema due to ClC-2 chloride channel deficiency: an observational analytical study. Lancet Neurol 12: 659–668. doi:10.1016/S1474-4422(13)70053-X

81. Di Bella D, Pareyson D, Savoiardo M, Farina L, Ciano C, Caldarazzo S, et al. (2014) Subclinical leukodystrophy and infertility in a man with a novel homozygous CLCN2 mutation. Neurology 83: 1217–1218. doi:10.1212/WNL.0000000000000812

82. Imagawa E, Osaka H, Yamashita A, Shiina M, Takahashi E, Sugie H, et al. (2013) A hemizygous GYG2 mutation and Leigh syndrome: a possible link? Hum Genet 133: 225–234. doi:10.1007/s00439-013-1372-6

83. Wang M, Wang JY, Cisler J, Imaizumi K, Burton BK, Jones MC, et al. (1997) Three novel fibrillin mutations in exons 25 and 27: classic versus neonatal Marfan syndrome. Hum Mutat 9: 359–362. doi:10.1002/(SICI)1098-1004(1997)9:4<359::AID-HUMU10>3.0.CO;2-1

84. Miller WL, Huang N, Pandey AV, Flück CE, Agrawal V (2005) P450 oxidoreductase deficiency: a new disorder of steroidogenesis. Ann N Y Acad Sci 1061: 100–108. doi:10.1196/annals.1336.012
