# Supplementary figures and images for: A Comprehensive Genomic Analysis Reveals the Genetic Landscape of Mitochondrial Respiratory Chain Complex Deficiencies
Source: PLoS Genet. 2016 Jan 7;12(1):e1005679. doi: 10.1371/journal.pgen.1005679 (PMC4704781; doi:10.1371/journal.pgen.1005679)

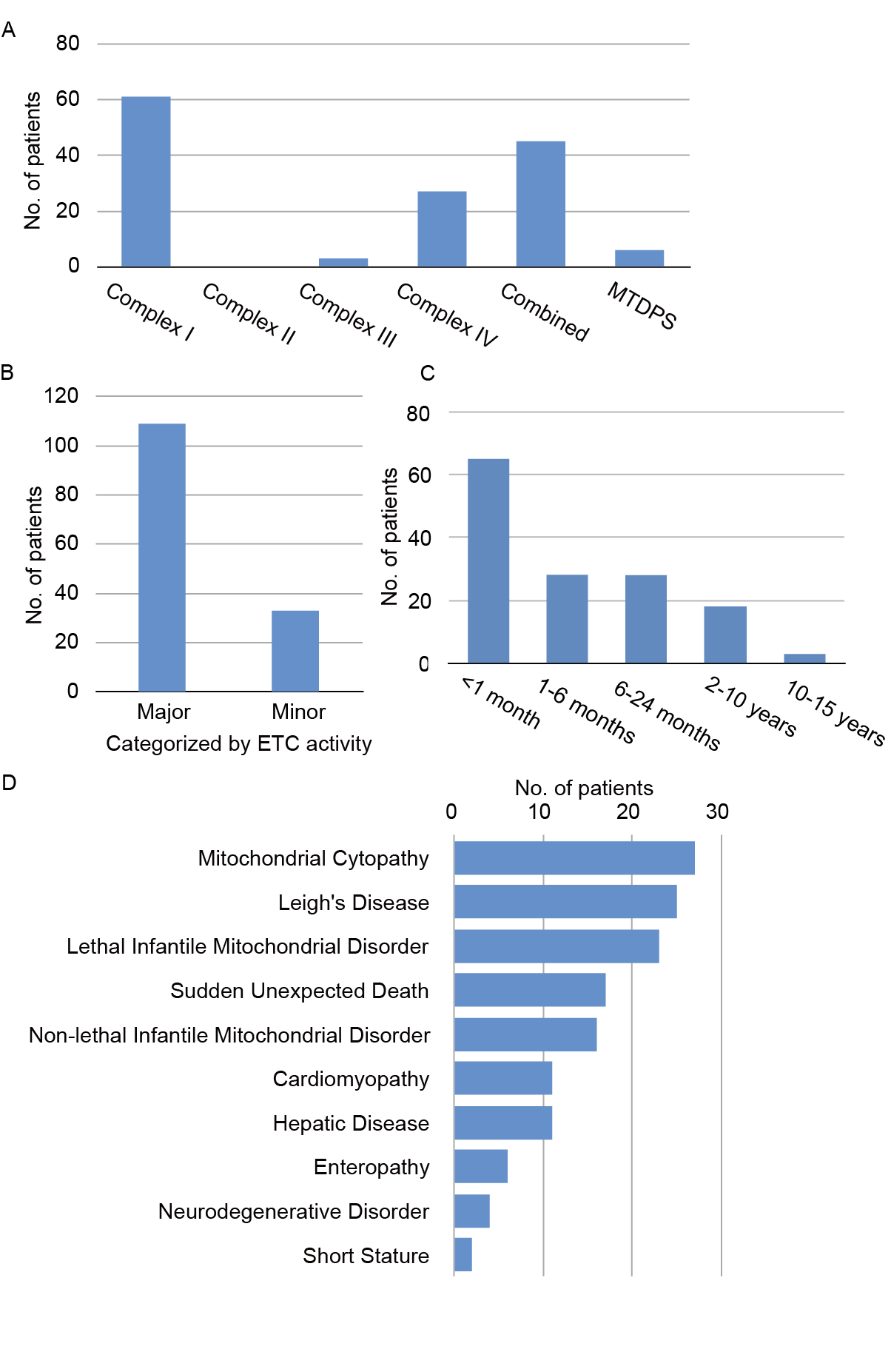

Supplement: S1 Fig — Histogram of enzymatic diagnoses in our cohort (A). Severity was categorized as “Major criteria” or “Minor criteria” on the basis of the level of enzyme activity reduction (B). Distribution of age at diagnosis (C). Histogram of clinical diagnoses in our cohort (D). MTDPS, mitochondrial DNA depletion syndrome; ETC, electron transport chain. (TIF) [file pgen.1005679.s002.tif]

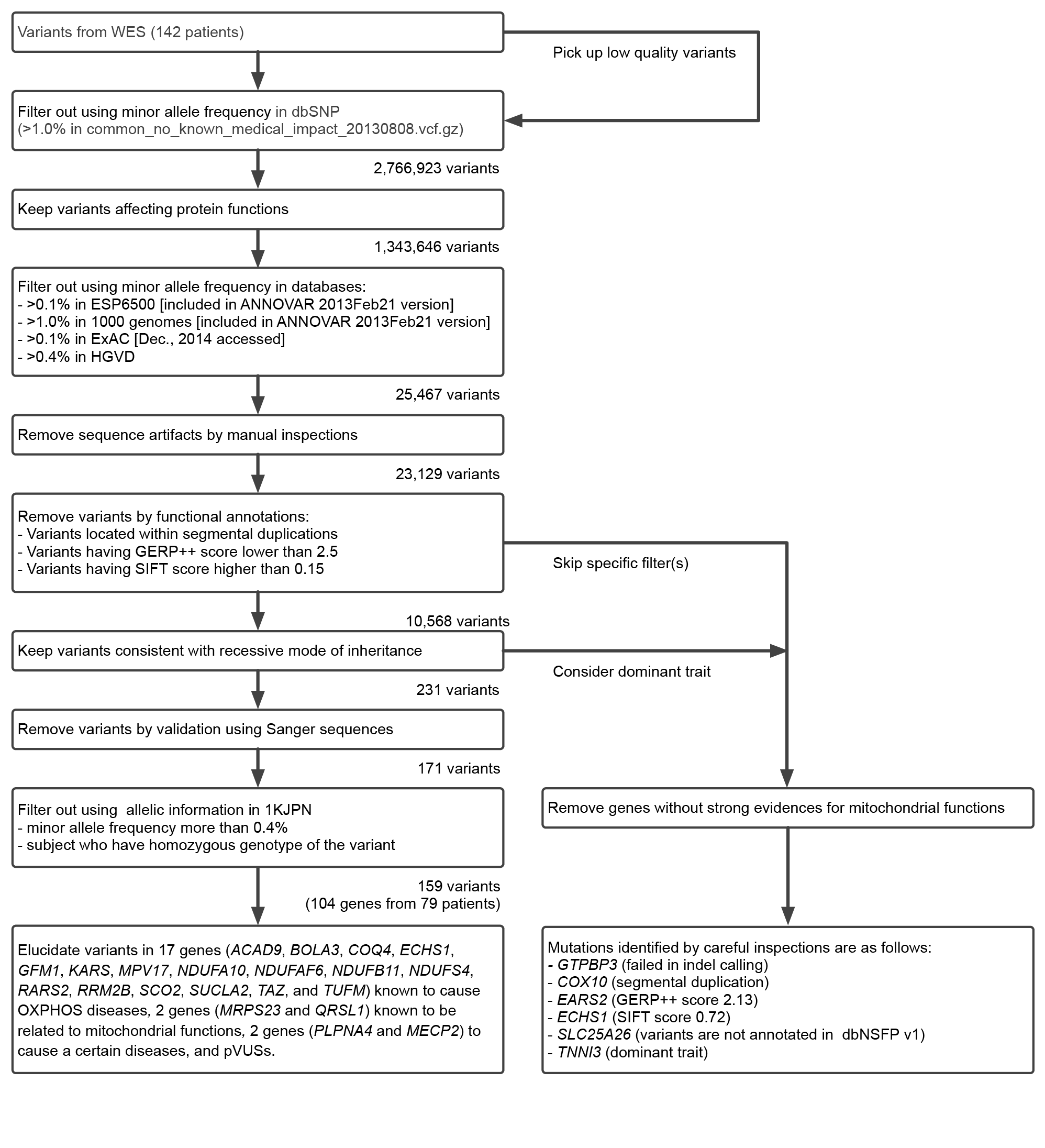

Supplement: S2 Fig — (TIF) [file pgen.1005679.s003.tif]

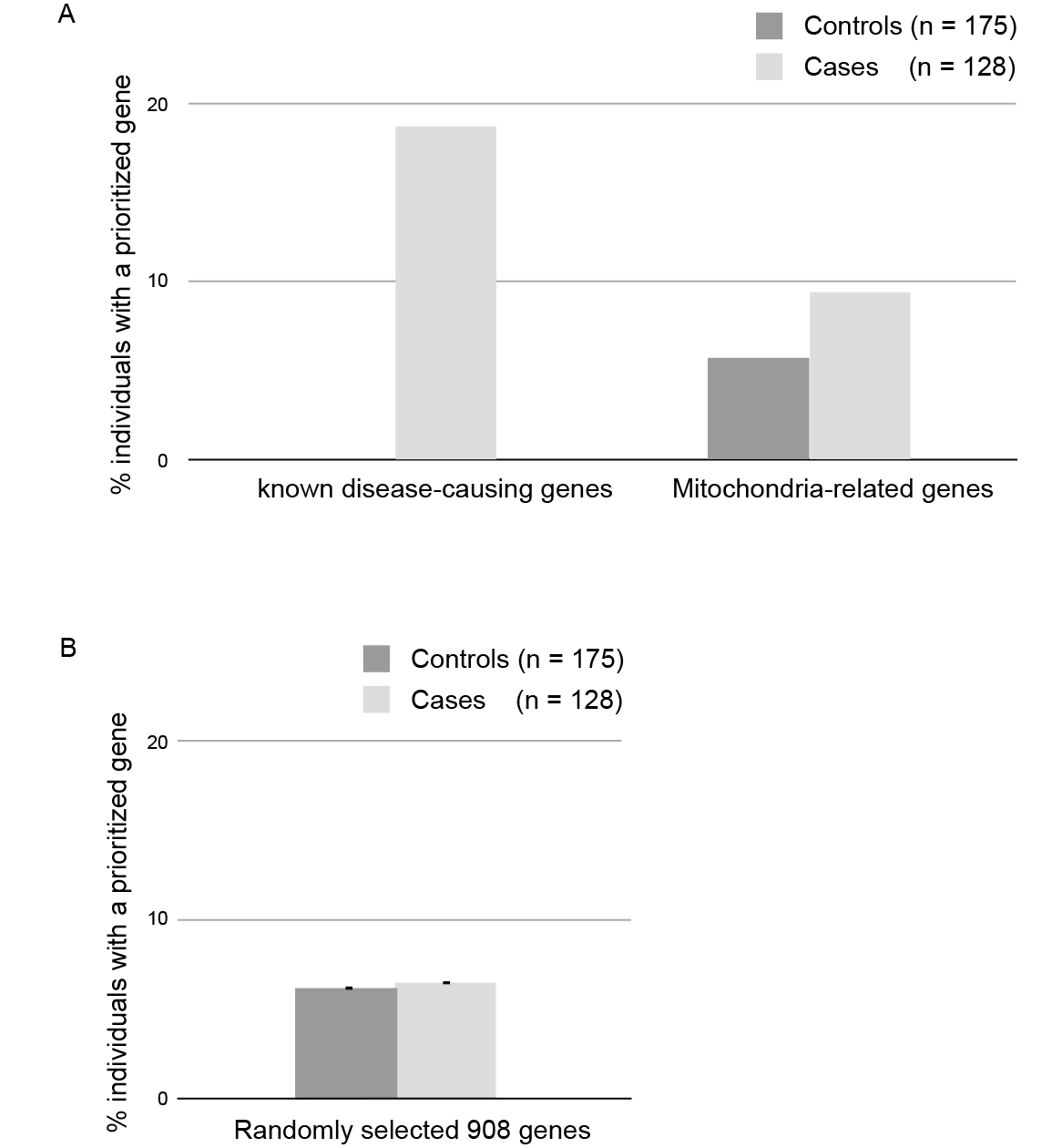

Supplement: S3 Fig — One hundred twenty-eight out of 142 cases, and 175 ethnically matched healthy controls whose sequence reads exceeded 50 million were included in the analysis. Percentages of cases and controls containing prioritized known OXPHOS disease-causing genes were elucidated using simplified exome analysis (A). Percentages of cases and controls containing prioritized mitochondria-related genes were elucidated using simplified exome analysis (A). Percentage of cases and controls containing 908 randomly selected other genes were elucidated using simplified exome analysis (B). (TIF) [file pgen.1005679.s004.tif]

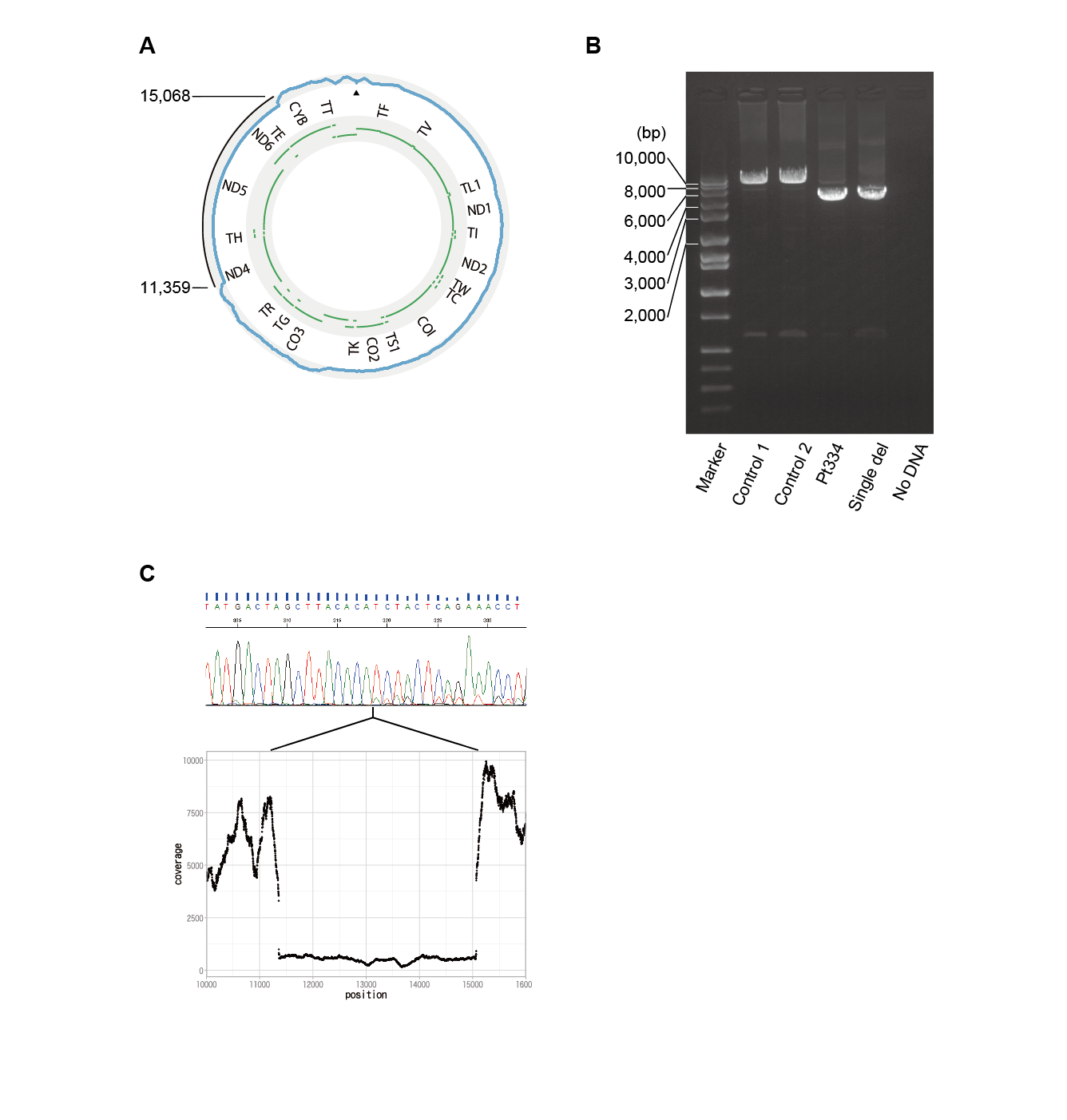

Supplement: S4 Fig — Schematic diagram of mtDNA indicates the deletion m.11359_15068del3710 (black arc) (A). Gel electrophoresis (1% agarose gel) of a long-range PCR amplicon shows an 8,701 bp fragment in control DNA and 4,992-bp fragment in Pt334; All Purpose Lo DNA Marker (BNX) and mtDNA with a deletion from other patient (Single del) are also included (B). mtDNA sequence coverage (C). Inset shows Sanger electropherogram of the breakpoint. (TIF) [file pgen.1005679.s005.tif]

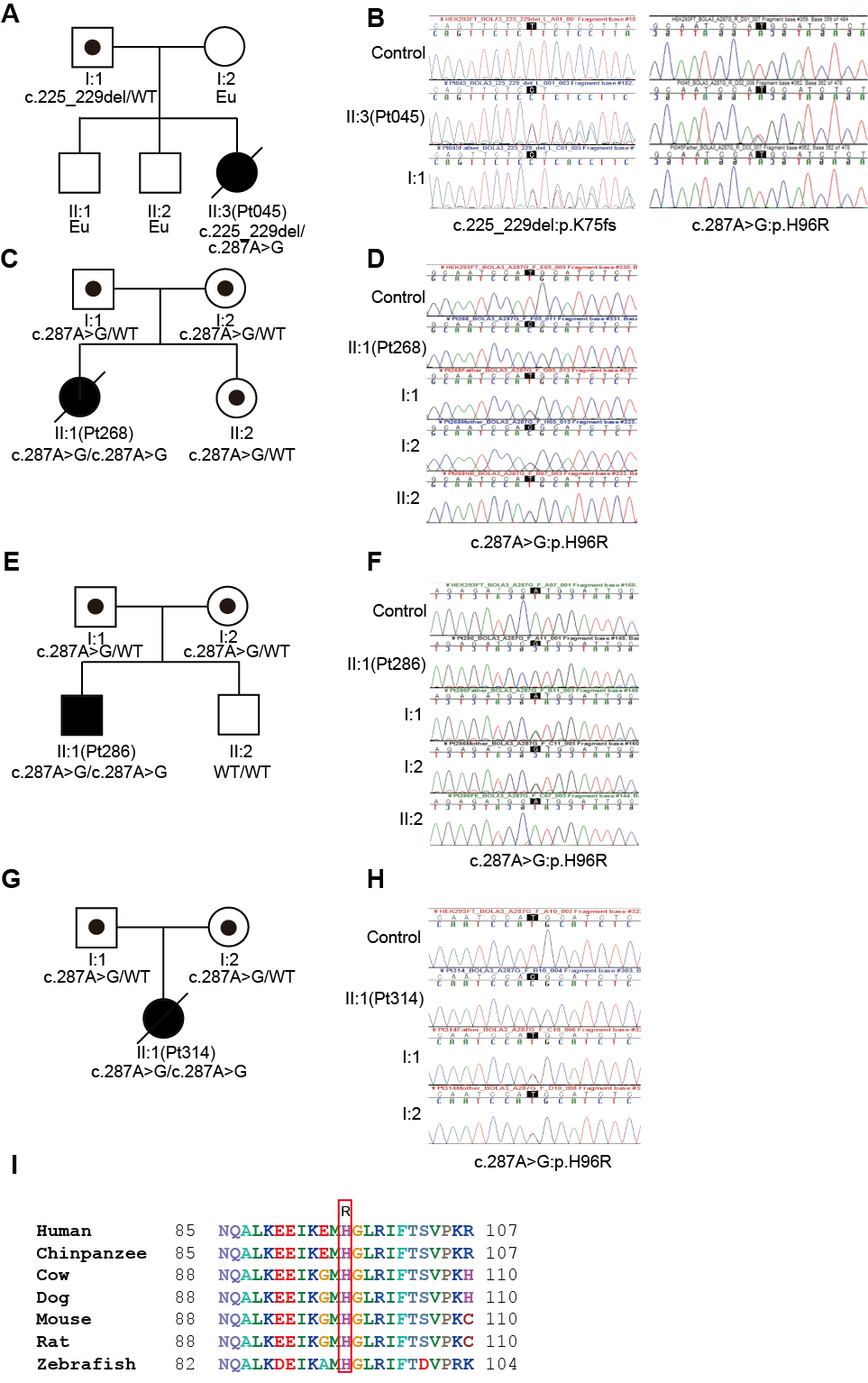

Supplement: S5 Fig — Family pedigrees and Sanger sequencing results for Pt045 (A and B), Pt268 (C and D), Pt286 (E and F), and Pt314 (G and H). Compound heterozygous mutations c.287A>G (p.H96R) and c.225_229del (p.K75fs) in BOLA3 (NM_212552) were identified in Pt045. Pt268, Pt286, and Pt314 had a homozygous mutation c.287A>G (p.H96R) in BOLA3 (NM_212552). Eu means uninformative DNA test. ClustalW alignment of BOLA3 orthologs. The residue p.H96 is highly conserved (I). (TIF) [file pgen.1005679.s006.tif]

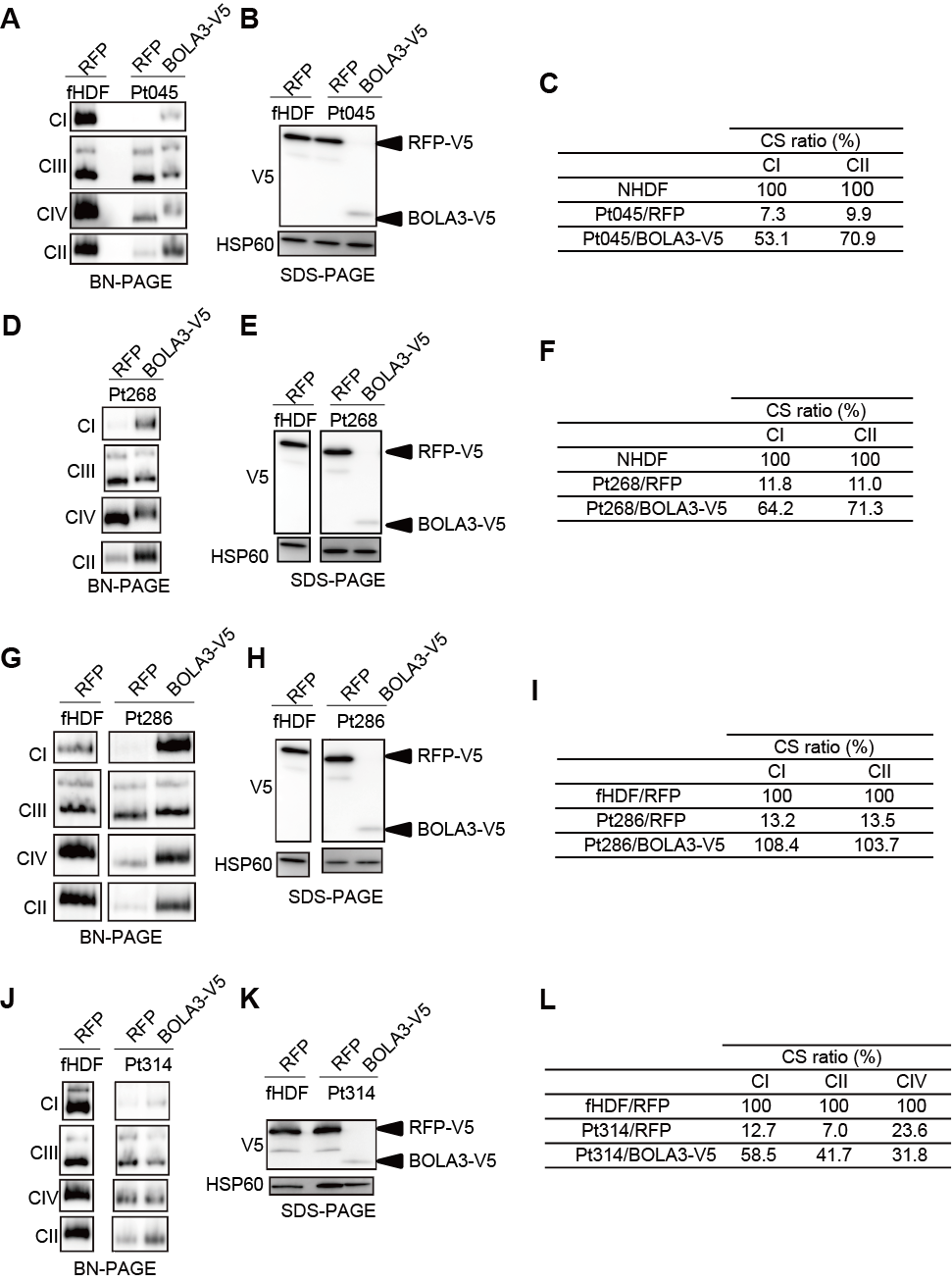

Supplement: S6 Fig — The mitochondria were isolated from control or patient fibroblasts following the lentiviral-mediated expression of mito-TurboRFP-V5 or BOLA3-V5 cDNA and were analyzed by BN-PAGE/Western blotting and mitochondrial respiratory chain complex enzyme assays. Complementation with BOLA3-V5 restored the assembly levels of both complexes (A,D,G,J) and enzyme activities (C,F,I,L) in all patient fibroblasts. mito-TurboRFP-V5 and BOLA-V5 proteins in the isolated mitochondria were detected by SDS-PAGE/Western blotting (B,E,H,K). HSP60 was used as a loading control. RFP, mito-TurboRFP-V5; CS, citrate synthase. (TIF) [file pgen.1005679.s007.tif]

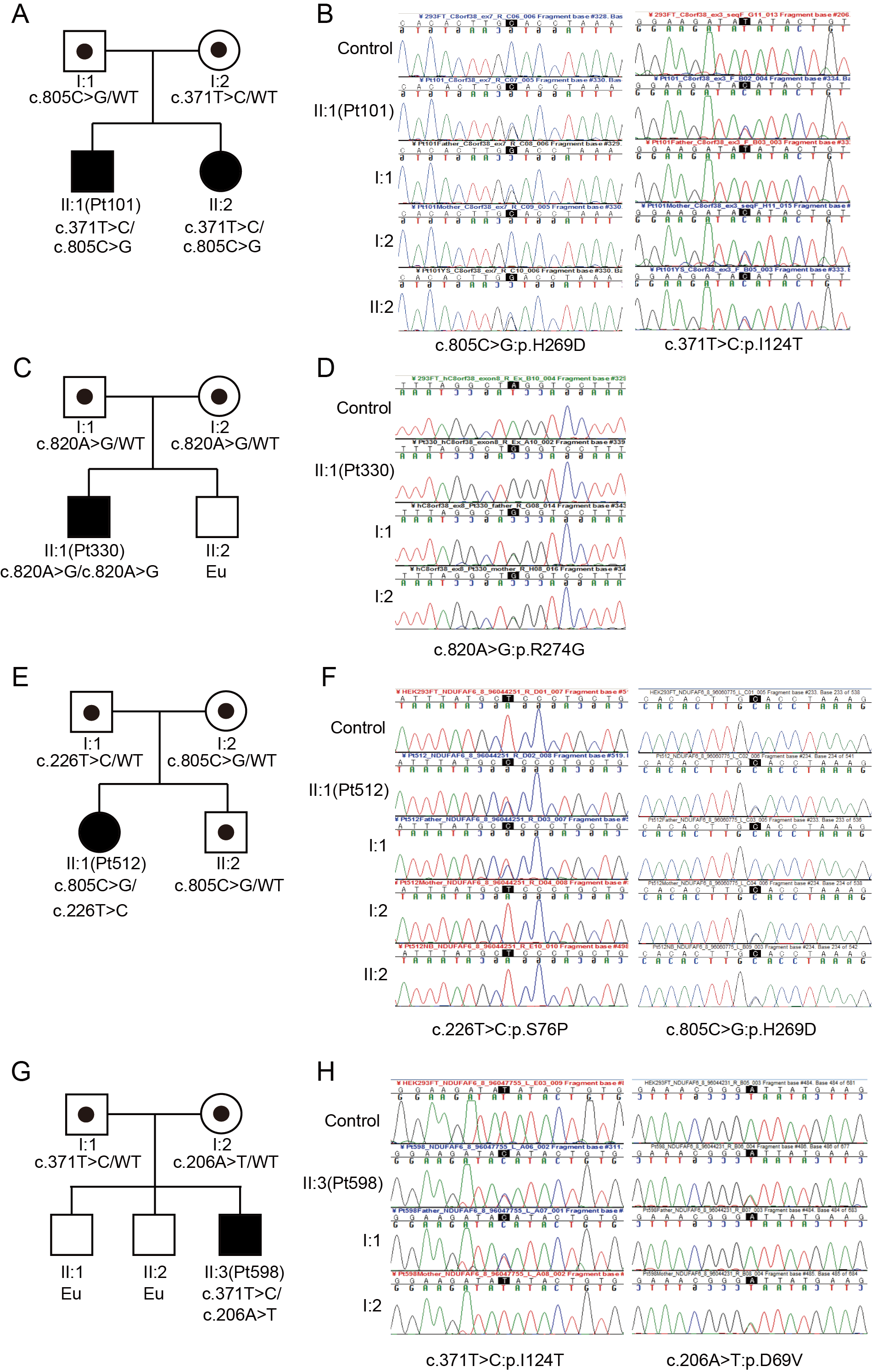

Supplement: S7 Fig — Family pedigrees and electropherograms for Pt101 (A and B), Pt330 (C and D), Pt512 (E and F), and Pt598 (G and H). The mutation c.371T>C in NDUFAF6 (NM_152416) was shared between Pt101 and Pt598. The mutation c.805C>G was found in both Pt101 and Pt512. Eu means uninformative DNA test. (TIF) [file pgen.1005679.s008.tif]

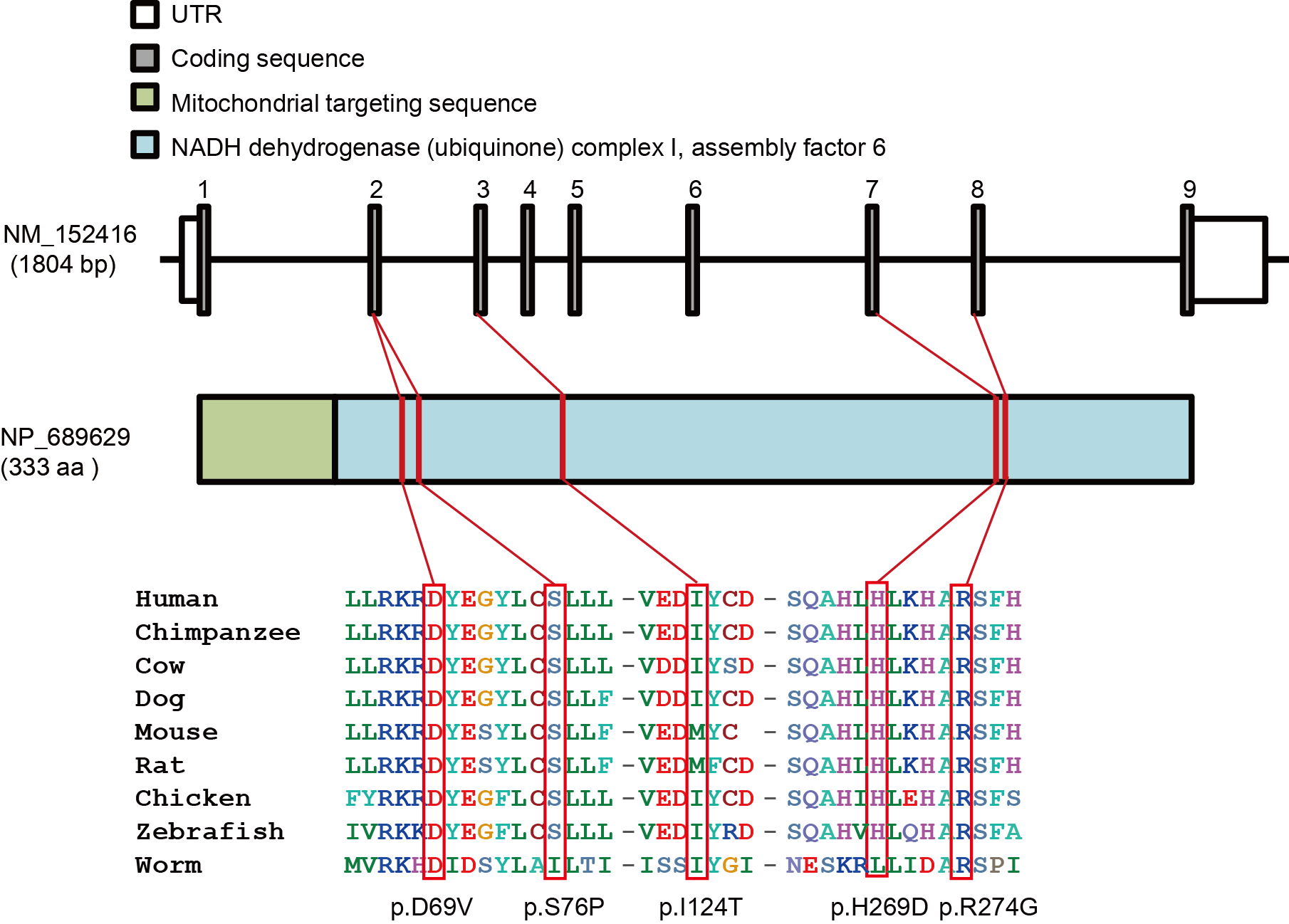

Supplement: S8 Fig — (TIF) [file pgen.1005679.s009.tif]

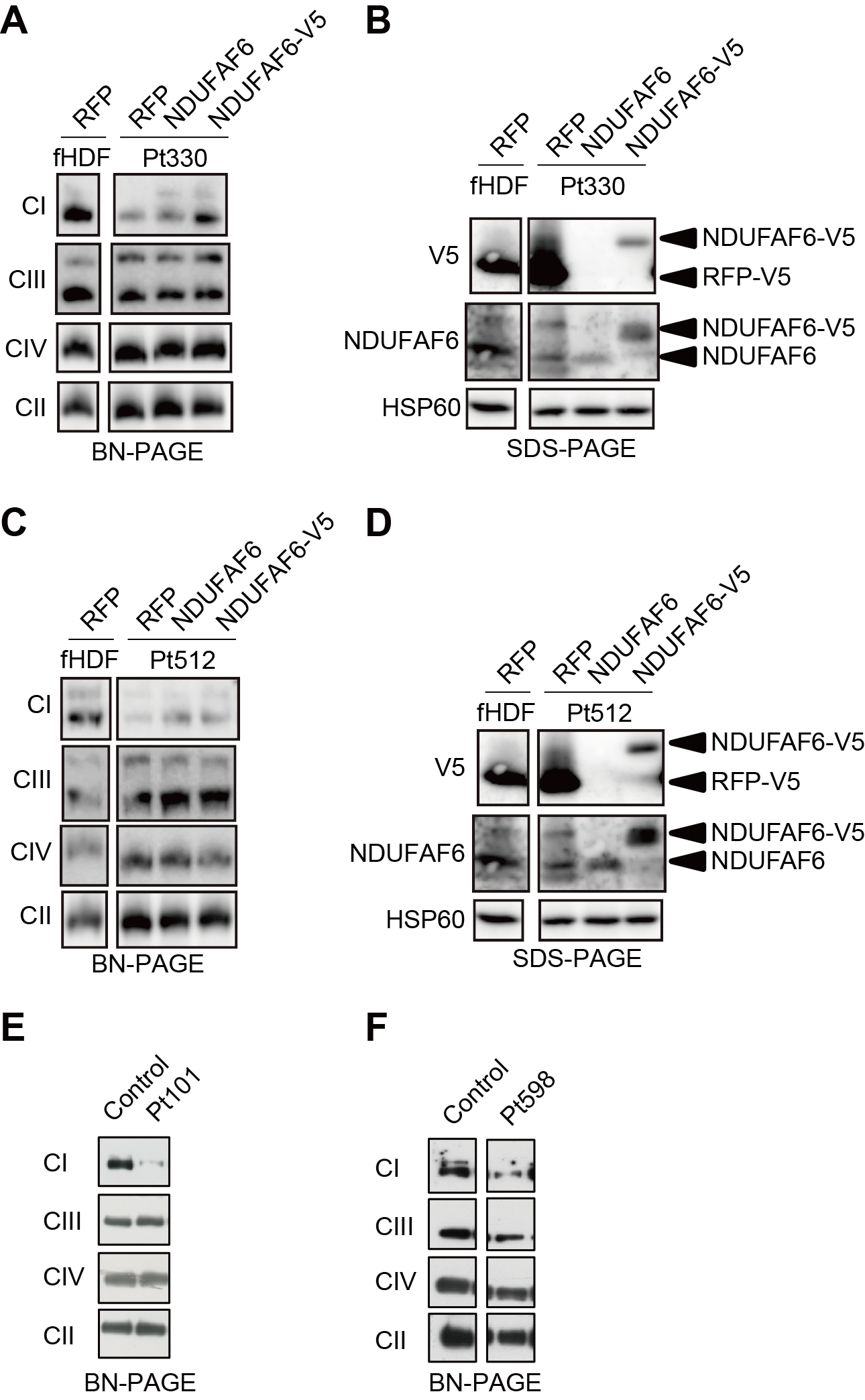

Supplement: S9 Fig — The mitochondria were isolated from control fibroblasts or Pt330 and Pt512 fibroblasts following the lentiviral-mediated expression of mito-TurboRFP-V5, NDUFAF6, or NDUFAF6-V5 cDNA and were analyzed by BN-PAGE and Western blotting. Complementation with NDUFAF6 or NDUFAF6-V5 restored the assembly levels of complex I in patient fibroblasts (A and C). mito-TurboRFP-V5, NDUFAF6 and NDUFAF6-V5 proteins in the isolated mitochondria were detected by SDS-PAGE/Western blotting with anti-V5 and anti-NDUFAF6 antibodies (B and D). HSP60 was used as a loading control. RFP, mito-TurboRFP-V5. BN-PAGE of mitochondrial fractions from Pt101 and Pt598 fibroblast cells (E and F). (TIF) [file pgen.1005679.s010.tif]

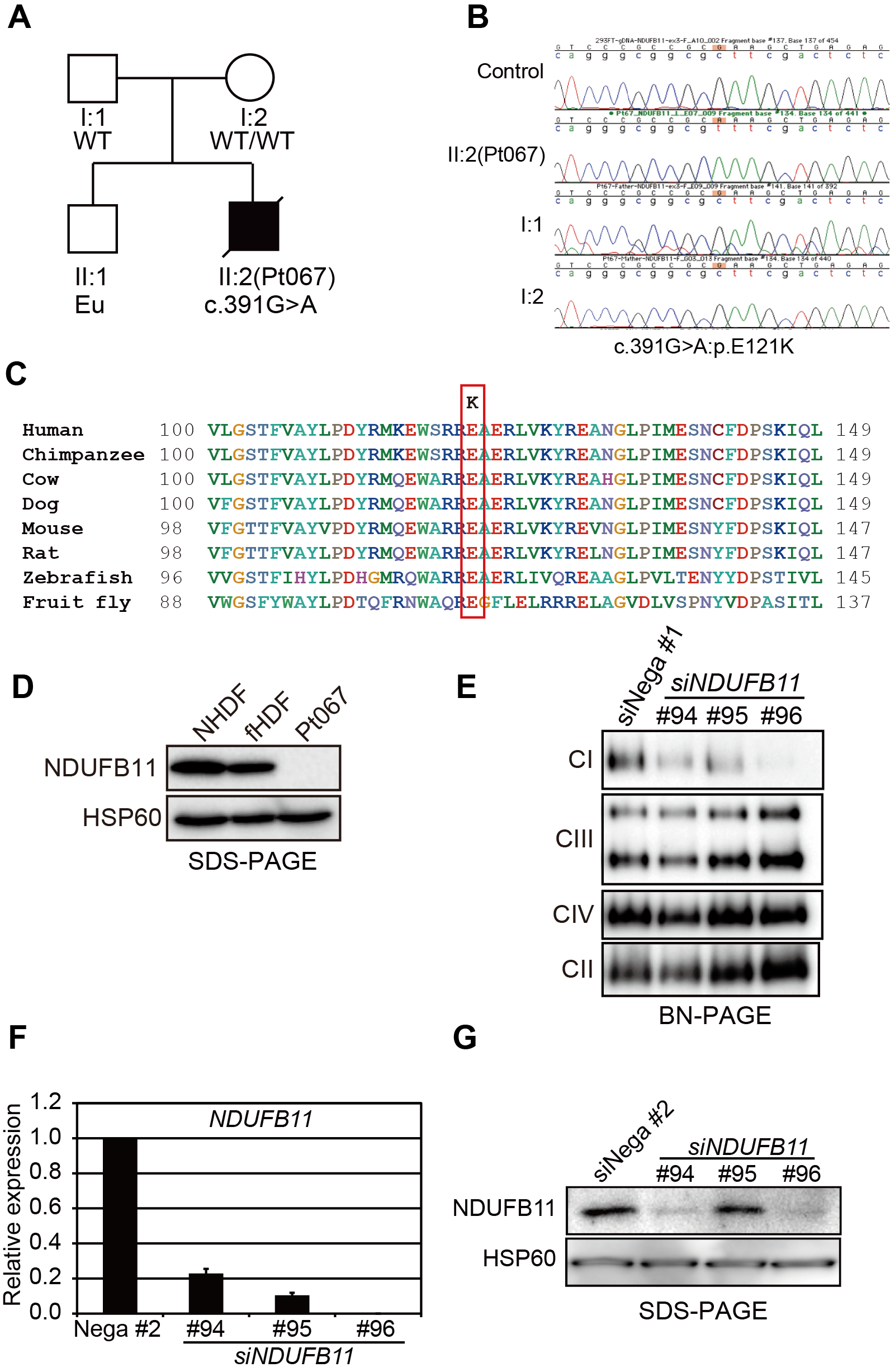

Supplement: S10 Fig — Family pedigrees of Pt067. The hemizygous c.391G>A mutation was de novo (A). Electropherograms for Pt067 (B). A hemizygous mutation c.361G>A (p.E121K) in NDUFB11 (NM_001135998) was found in Pt067. Eu means uninformative DNA test. ClustalW alignment of NDUFB11 orthologs shows conservation of the p.E121 residue (C). SDS-PAGE/Western blotting analysis showed a decrease in the endogenous NDUFB11 protein level in Pt067 fibroblasts. HSP60 was used as a loading control (D). siRNA knockdown of NDUFB11 in normal fibroblasts. BN-PAGE/Western blotting of mitochondrial fractions derived from normal human fibroblasts transfected with siNDUFB11 (E). NDUFB11 mRNA and NDUFB11 protein expression in cells treated with siRNAs targeting NDUFB11 (Clone #94, #95 and #96) and control cells (siNega #2) were confirmed by quantitative real-time PCR and SDS-PAGE/Western blotting, respectively (F and G). (TIF) [file pgen.1005679.s011.tif]

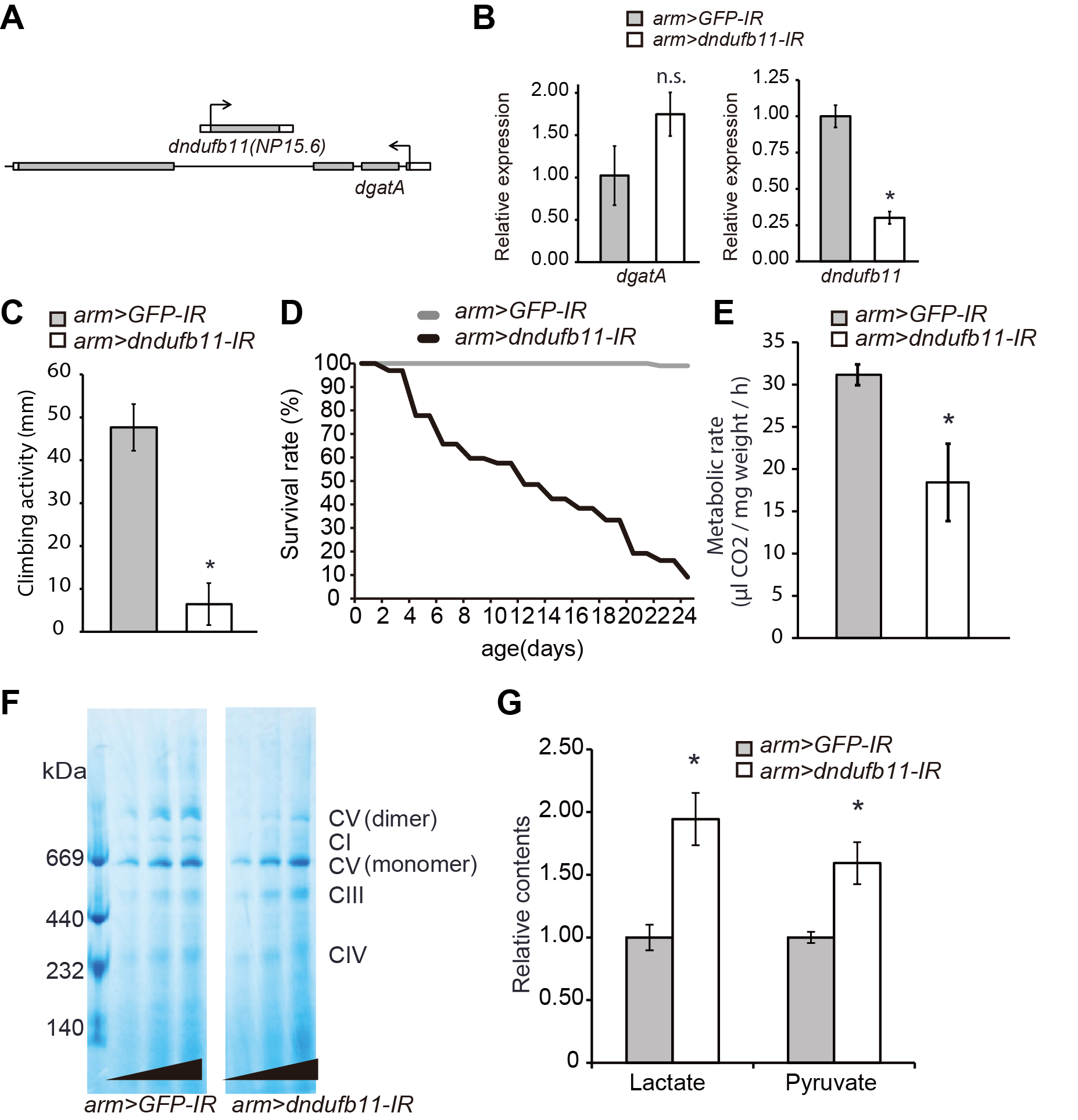

Supplement: S11 Fig — Schematic representation of dndufb11 (NP15.6) and dgatA in Drosophila melanogaster (A). dndufb11 is located in intron 3 of dgatA, a QRSL1 ortholog. Quantitative PCR revealed dramatically reduced dndufb11 mRNA levels in RNAi-generated dndufb11-knockdown flies (arm > dndufb11-IR) to approximately 24% of control levels (arm > GFP-IR), whereas dgatA mRNA levels were normal (B). All relative values were calculated against data from control flies. Climbing activities of arm > dndufb11-IR flies and control flies at the age of 6 days (C). Significance relative to controls was calculated using Student’s t test (*; p < 0.01; n.s., not significant). The longevity of arm>dndufb11-IR male flies was dramatically reduced relative to that of control flies (log-rank test; p < 0.01) (D). Metabolic rate was estimated from CO2 production (μl CO2/mg weight/h) of 4-day-old male flies (E). BN-PAGE of mitochondrial fractions from arm>dndufb11-IR and control male flies (F). The mitochondria were resuspended in native-PAGE sample buffer with 1% digitonin. Wedges indicate increasing amounts of mitochondrial extract (10 μg, 25 μg, 50 μg). Relative amounts of lactate and pyruvate in dndufb11-knockdown flies against control flies (G). The amounts of metabolites were determined by LC-MS analysis. Data represent mean ± SEM of at least three experiments. Student's t-test was used to compare the data between dndufb11-knockdown and control (arm>GFP-IR) flies. (*; p < 0.01). (TIF) [file pgen.1005679.s012.tif]

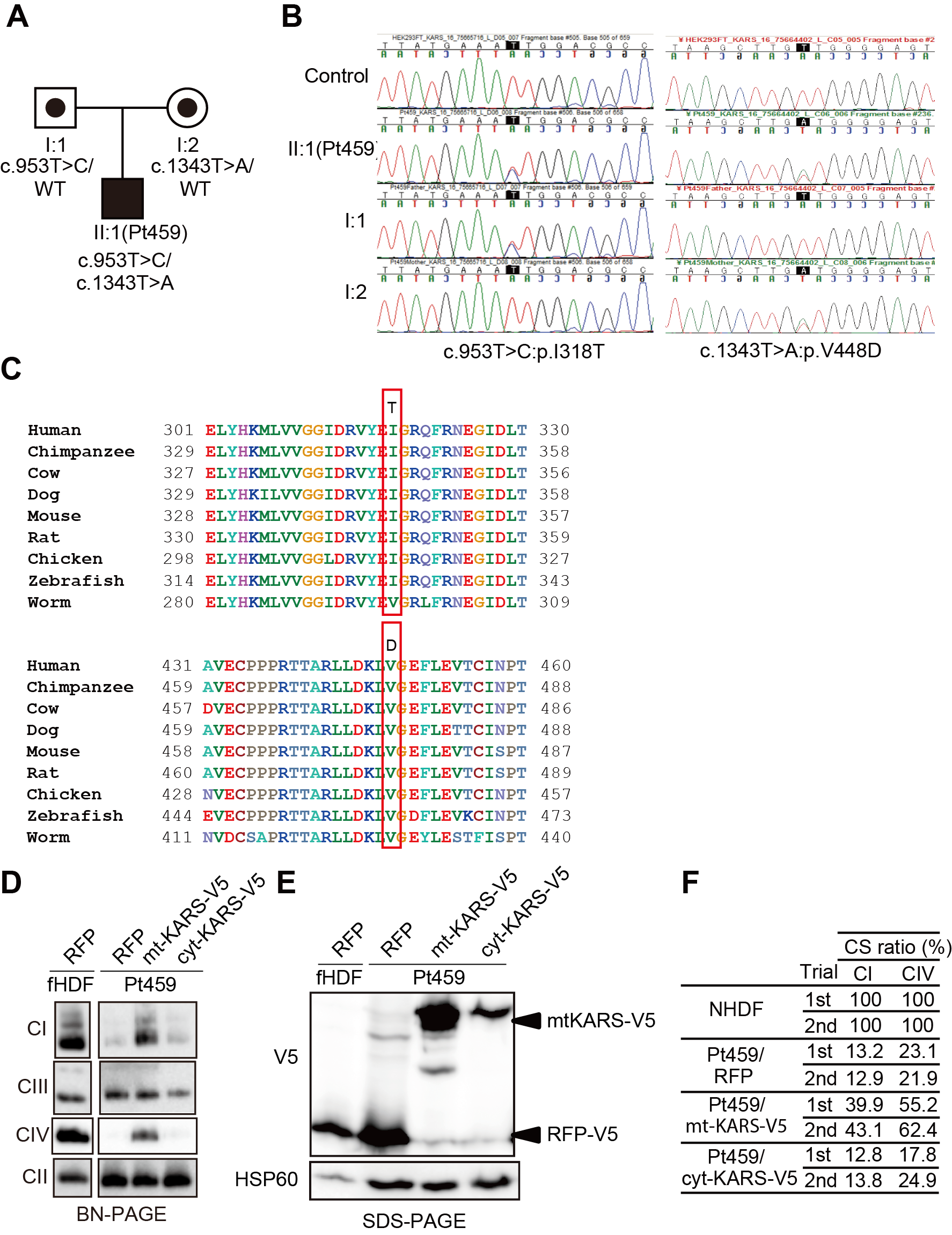

Supplement: S12 Fig — Family pedigrees of Pt459 (A). Electropherograms for Pt459 (B). Compound heterozygous mutations c.1343T>A (p.V448D) and c.953T>C (p.I318T) in KARS (NM_005548) found in Pt459. ClustalW alignment of KARS orthologs shows conservation of both the p.V448 and p.I318 residues (C). mito-TurboRFP-V5, mtKARS-V5 (mitochondrial isoform of KARS), and cytKARS-V5 (cytosolic isoform of KARS) proteins in isolated mitochondria were detected by SDS-PAGE/Western blotting with an anti-V5 antibody (E). HSP60 was used as a loading control. Respiratory chain complex enzyme activity was measured twice; activity is shown as a percentage of citrate synthase activity. Enzyme activities (D) and complex I and IV assembly (F) were rescued in Pt459 fibroblasts by the overexpression of wild-type mitochondrial KARS cDNA. RFP, mito-TurboRFP-V5; mt-KARS, mitochondrial KARS (NM_001130089)-V5; cyt-KARS, cytosolic KARS (NM_005548)-V5; CS, citrate synthase. (TIF) [file pgen.1005679.s013.tif]

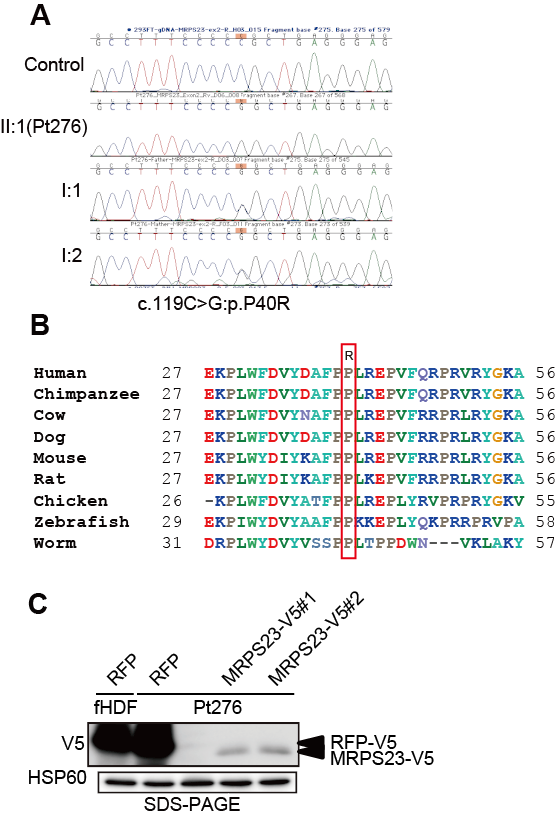

Supplement: S13 Fig — Electropherograms for Pt276 (A). A homozygous mutation c.119C>G (p.P40R) in MRPS23 (NM_016070) was found in Pt276. ClustalW alignment of MRPS23 orthologs shows conservation of the p.P40 residue (B). MRPS23-V5 protein expression in MRPS23-V5-overexpressing Pt276 cell lines was confirmed by SDS-PAGE/Western blotting with an anti-V5 antibody (C). RFP, mito-TurboRFP-V5. (TIF) [file pgen.1005679.s014.tif]

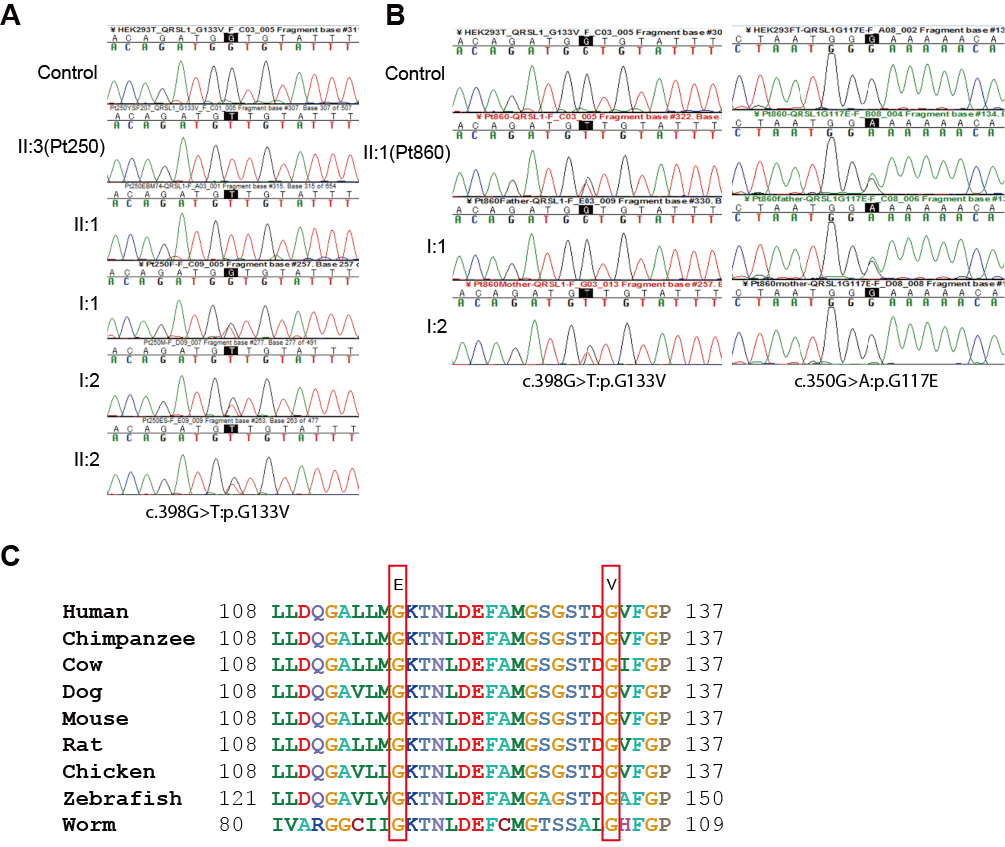

Supplement: S14 Fig — Electropherograms for Pt250 (A) and Pt860 (B). Homozygous and compound heterozygous mutations c.398G>T (p.G133V) and c.350G>A (p.G117E) in QRSL1 (NM_018292) found in Pt250 and Pt860, respectively. Sequence alignment of QRSL1 orthologs (C). (TIF) [file pgen.1005679.s015.tif]

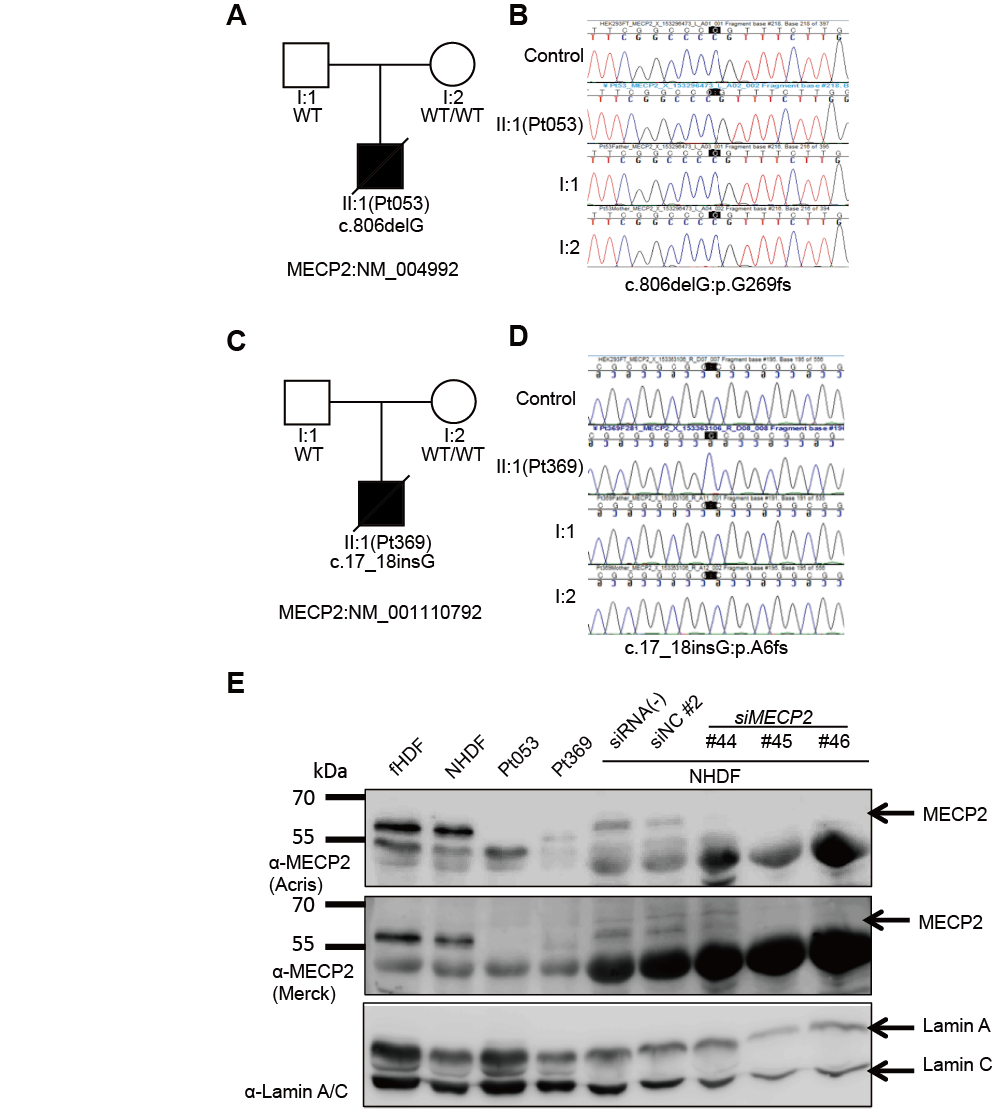

Supplement: S15 Fig — Family pedigree and electropherograms for Pt053 (A and B). The c.806delG (p.G269fs) mutation in MECP2 (NM_004992) has been reported in males with severe neonatal encephalopathy. Family pedigree and electropherograms for Pt369 (C and D). Another novel mutation in MECP2 (NM_001110792) c.17_18insG (p.A6fs) was identified in Pt369. SDS-PAGE/Western blotting analysis exhibited loss of the endogenous MECP2 protein level in isolate nuclear fractions from Pt053 and Pt369 fibroblasts and MECP2 knockdown fibroblasts (E). Lamin A/C was used as a loading control. (TIF) [file pgen.1005679.s016.tif]

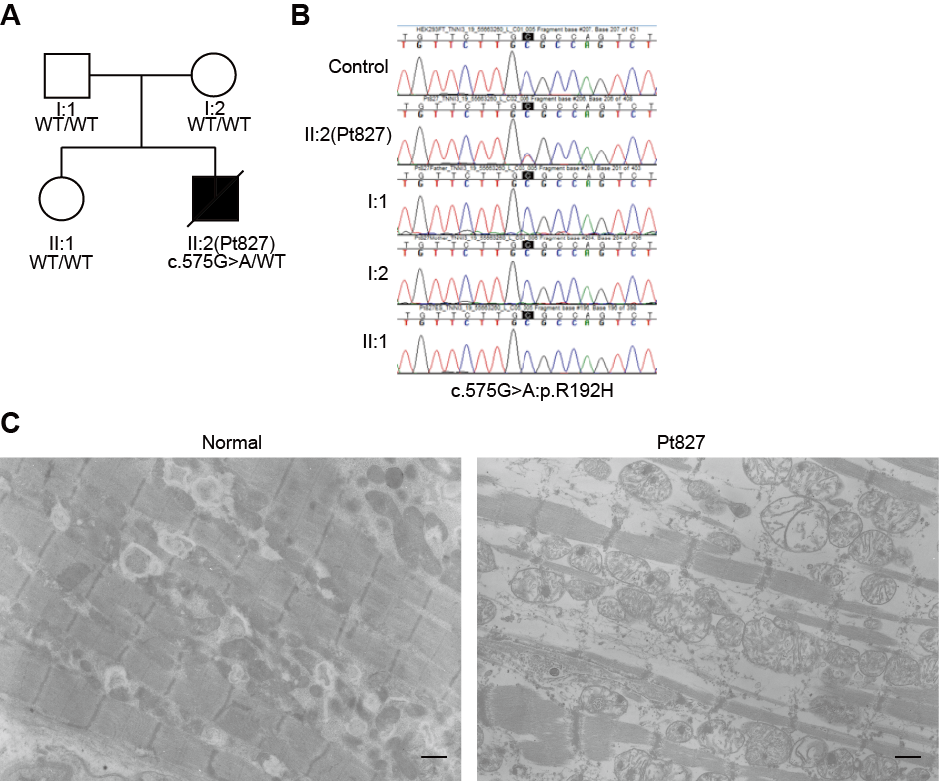

Supplement: S16 Fig — Family pedigree for Pt827 (A and B). A heterozygous mutation c.575G>A (p.R192H) in TNNI3 (NM_000363), which was previously identified in patients with restrictive cardiomyopathy, was confirmed de novo via Sanger sequencing of the parents’ DNA. The transmission electron microscope image of the mitochondria in a cardiac muscle tissue of normal individual and the patient (C). The mitochondria were often enlarged with increased cristae density. Scale bar, 500 nm. (TIF) [file pgen.1005679.s017.tif]

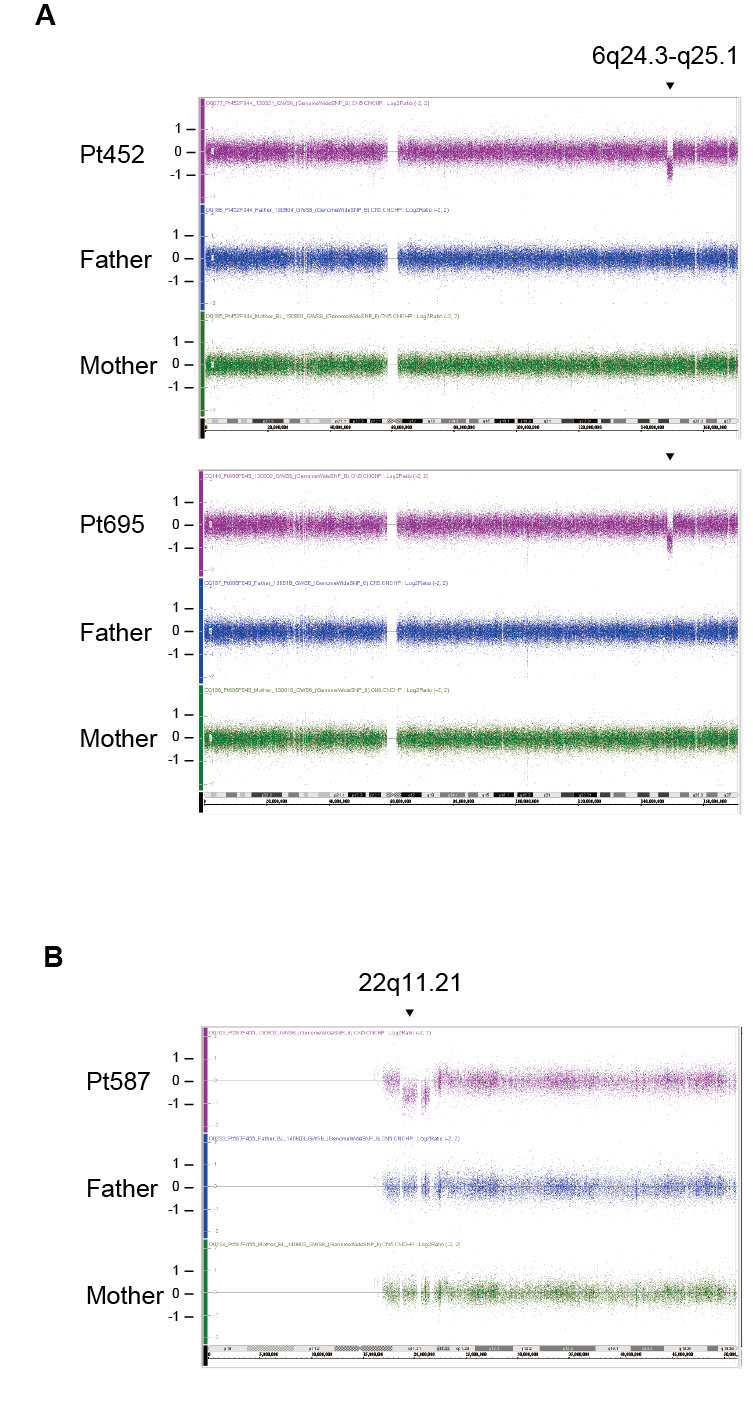

Supplement: S17 Fig — De novo deletions of 6q24.3-q25.1 were identified using a high-density oligonucleotide array in Pt452 and Pt695 (A). De novo deletion of 22q11.21 was identified using a high-density oligonucleotide array in Pt587 (B). (TIF) [file pgen.1005679.s018.tif]
